# Programmable nanocarriers for precision plant engineering: converging nanotechnology, CRISPR, and next-generation breeding

**DOI:** 10.3389/fpls.2026.1845328

**Published:** 2026-06-05

**Authors:** Aditya Pratap Singh, Saba Haider, Ashutosh Sawarkar, Sanjivani Karki, Kousik Atta, Mohammed Al-zharani, Jayanthi Barasarathi, Nazih Y. Rebouh, Zienab F. R. Ahmed

**Affiliations:** 1 Department of Genetics and Plant Breeding, Bidhan ChandraKrishi Viswavidyalaya, Kalyani, West Bengal, India; 2 Department of Horticulture, PMAS-Arid Agriculture University, Rawalpindi, Pakistan; 3 Department of Genetics and Plant Breeding, IRDM Faculty Centre, Ramakrishna Mission Vivekananda Educational and Research Institute (RKMVERI), Narendrapur, Kolkata, India; 4 Department of Agronomy, Uttar Banga Krishi Viswavidyalaya, Coochbehar, India; 5 Faculty of Agricultural Sciences, GLA University, Mathura, India; 6 Biology Department, College of Science, Imam Mohammad Ibn Saud Islamic University (IMSIU), Riyadh, Saudi Arabia; 7 Faculty of Health & Life Sciences (FHLS), INTI International University, Nilai, Negeri Sembilan, Malaysia; 8 Institute of Environmental Engineering, RUDN University, Moscow, Russia; 9 Integrative Agriculture Department, College of Agriculture and Veterinary Medicine, United Arab Emirates University, Al Ain, United Arab Emirates

**Keywords:** CRiSPR/Cas, nano–bio interface, plant genome editing, plant transformation, precision breeding, regulatory harmonization, transgene-free crops

## Abstract

The convergence of nanotechnology and genome editing in plant sciences is redefining modern precision breeding through efficient, transgene free, tissue culture independent pathways for genetic improvement in crops. Conventional breeding and transgenic tools are limited to genotype dependency, inefficient gene delivery, unpredictable transgene insertions, thereby restricting their application in elite germplasm. Nanoparticles-mediated gene delivery systems have revolutionized the genetic transformation in plants through targeted and transgene free delivery of CRISPR/Cas ribonucleoproteins (RNPs), DNA, and RNA into plant cells, while minimizing genome interference. Nanocarriers are the engineered delivery systems wherein the material component is a nanoparticle. DNA-free delivery refers to the absence of exogenous DNA during editing, whereas transgene free plants are those that do not retain integrated foreign DNA after regeneration. Firstly, this review summarizes current progress in designing nanocarriers, including lipid, polymeric, mesoporous silica nanoparticles, carbon-based nanoparticles, layered double hydroxides, and DNA-based nanoparticles; harnessing the function of their physicochemical traits in modulating plant cellular uptake, cargo stability, controlled delivery, and tissue specific targeting in plants. Secondly, the broad-spectrum roles of nano particles in genome editing, crop protection via RNA interference, organelle-targeted modifications are discussed, stressing transgene free approaches to mitigate somaclonal variation and regulatory concerns to foster public acceptance. The integration of nano-mediated delivery with speed breeding, meristem transformation, multiplexed editing in elite germplasm is proposed as an approach for prompt trait stacking and validation. Thirdly, the collaborative roles of experts in the field of nanotechnology, plant breeding, plant physiology, and agronomy are mentioned for mitigating multifaceted climatic effects and glitches. Moreover, current challenges including nanotoxicity, scalability and field translation, regulatory concerns, and public perception are also discussed. While nanocarrier mediated delivery shows strong potential for improving plant genome engineering, current evidence is largely confined to controlled experimental systems, and significant challenges remain before routine integration into breeding pipelines becomes feasible.

## 1 Introduction

### 1.1 Nanotechnology–genome editing convergence in plant science

Feeding a projected 9-10 billion people by 2050 under less available land, water, and climate changes will need global food production to be increased by 50-70% compared to 2005-2007 levels. However, the yields of several major crops are already reaching a ceiling, and new abiotic stresses, pests and pathogens that evolve, and the degradation of soils are gradually lowering the performance of existing varieties (van Dijk et al., 2021). The time from trait discovery to cultivar release in conventional breeding and transgenic pipelines is usually 10-15 years; even with genomic selection and speed breeding, the rate of genetic change is often slower than the rate of environmental change. As a result, genome editing with high precision is being hailed as the main crop improvement method of the future (Chen et al., 2024; Xie et al., 2022).

### 1.2 Limitations of conventional delivery and transformation systems

CRISPR–Cas systems have been successfully used to intervene in a range of crops, the genes responsible for yield, architecture, resource-use efficiency, and resistance to biotic and abiotic stresses can be specifically targeted and modified (Chen et al., 2024; Xie et al., 2022). Different traits edited by the system are reviewed in the literature, which include increased grain number and improved plant architecture in cereals, disease resistance conferred by loss-of-function in susceptibility genes, enhanced stress tolerance, and nutritional quality (Chen et al., 2024; Xie et al., 2022). Besides these, base and prime editors further broaden this toolkit by allowing highly accurate nucleotide changes and small insertions without the need for the introduction of double-strand breaks and are already being used in rice, wheat, and maize for herbicide tolerance, micronutrient biofortification, and stress resilience (Vats et al., 2024). Theoretically, these tools alone could shorten breeding cycles to a great extent, hence, breeders could effectively re-design elite varieties of their choice instead of being heavily dependent on recombination and selection.

Genome engineering in plants is a prime example where a single bottleneck. In this case, delivery of the editing agents to the right cells of the elite, often transformation-recalcitrant germplasm, thus without creating foreign DNA still throttles the technology drastically (Nivya et al., 2023). Insertion of foreign DNA through an Agrobacterium-mediated transformation is still the major method of delivery in a lot of crops due to its relatively low copy number integration and stable expression. However, it is very genotype-dependent and limited in the host range (Nivya et al., 2023; Kour et al., 2024). In principle, Biolistic (gene-gun) delivery can be used to transform a wider range of species and tissues, but it results in extensive DNA fragmentation, complex and multi-copy insertions, and tissue damage. On the one hand, PEG-mediated uptake into protoplasts and on the other hand, electroporation of embryogenic tissues, can circumvent some of these problems, but they require tedious, genotype-sensitive regeneration protocols and are not very scalable for elite breeding lines. Viral vectors provide high transient expression but their cargo capacity is limited and they are host range and regulatory restrictions (Chen et al., 2024; Xie et al., 2022).

These delivery limitations conflict directly with regulatory and public acceptance transgene-free edited crops plants that have small, precise sequence changes that are indistinguishable from spontaneously or chemically mutated ones (Chen et al., 2024; Vats et al., 2024). DNA-free methods using CRISPR ribonucleoprotein (RNP)complexes or transient expression of the editing machinery may, theoretically, produce such products, but they still depend on Agrobacterium, biolistics, or protoplast systems and therefore share genotype dependence, tissue culture bottlenecks, and somaclonal variation (Nivya et al., 2023; Xie et al., 2022). Consequently, the majority of the most complex edits that have been documented in the literature are limited to a few transformable model cultivars, while the most important germplasm for the world that is recalcitrant local landraces, regionally preferred mega-varieties, and clonally propagated crops are still largely inaccessible (Nivya et al., 2023).

### 1.3 Need for precision, transgene-free, and sustainable crop improvement

The emerging field of plant nanobiology over the last ten years indicates a major shift in the way genome engineering could be done. Particles of nanomaterials, which can have one dimension between 1 and 100 nm, can be created with controlled size, shape, surface charge, and chemistry, and these particles are now considered to be capable of crossing plant membranes that used to be regarded as impenetrable for biomolecule delivery (Cunningham et al., 2018).The primary cell wall has a size exclusion limit (SEL) of approximately 5–20 nm (Gao et al., 2023; Wang et al., 2023), however, several engineered nanomaterials especially high-aspect-ratio carbon nanotubes, small polymeric particles, and layered double hydroxide (LDH) nanosheets have been reported to pass through this barrier and penetrate the cells that make up the plant body via leaf, root, or pollen, respectively, without the type of physical damage that is normally associated with biolistics (Demirer et al., 2019). Inside the plant, nanoparticles can move through apoplast and symplast, be loaded into vascular tissues, and in a few instances, they can even be found in the organelles of particular cells, thus, the control over the delivery of the cargo in terms of space and time is at a level which has never been achieved before (Cunningham et al., 2018; Gao et al., 2023).

**Figure 1:**
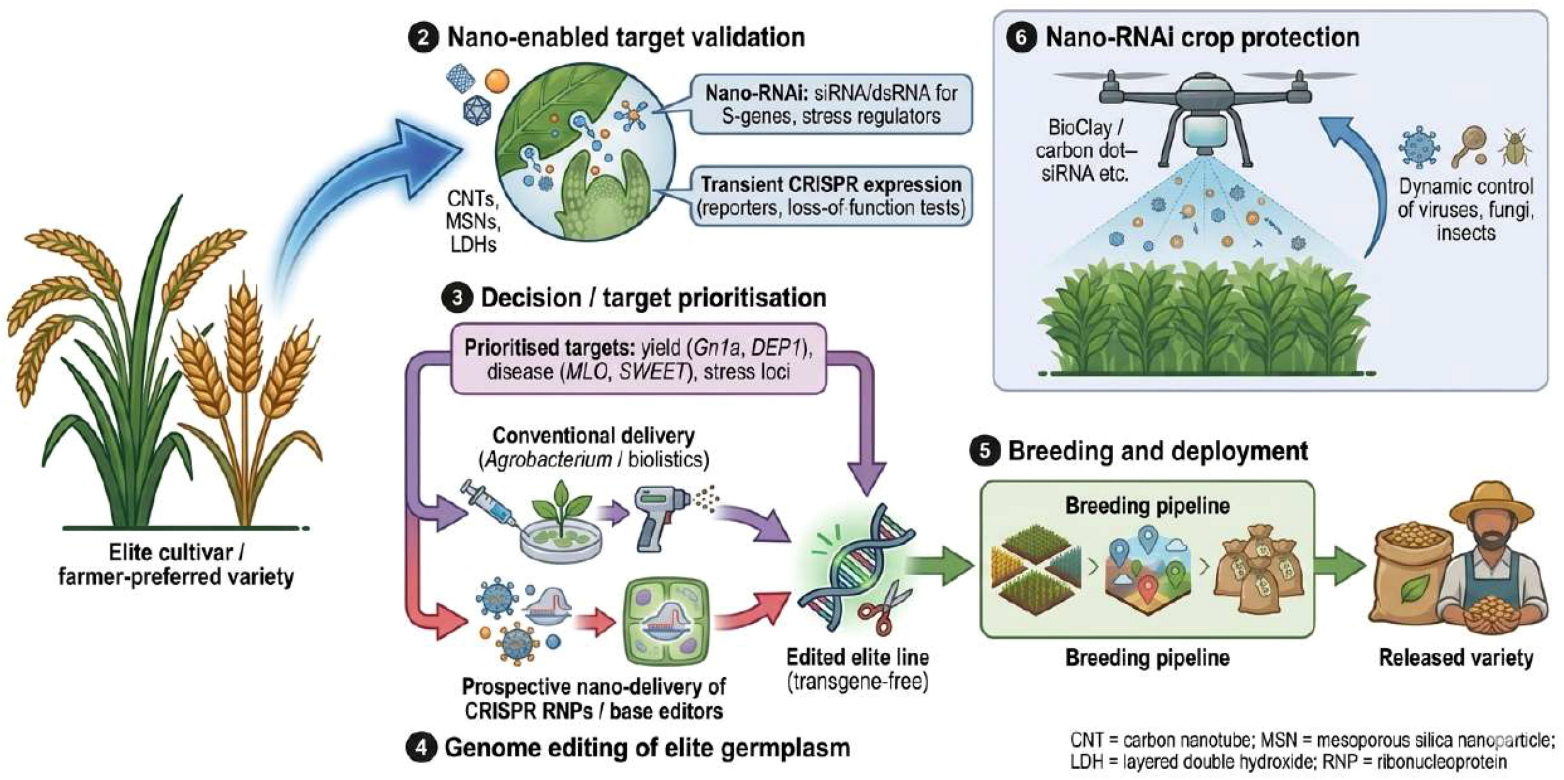
Integration of nanotechnology in crop breeding and protection pipelines. This diagram outlines a workflow from target identification in elite cultivars to the final release of improved varieties.

A number of landmark studies have, in essence, demonstrated how nucleic acids and proteins can be delivered into mature plants by means of nanoparticles. Carbon nanotubes of high aspect ratio and suitably polymer-functionalized can carry plasmid DNA into the leaves of different species, thus causing transient expression to a very high degree and without any genomic integration being detected (Demirer et al., 2019). The same and similar platforms have been modified to intracellularly transport siRNAs to achieve high silencing efficiencies while also maintaining the RNA sorption safe from nuclease degradation (Zhang et al., 2019). LDH “BioClay” nanosheets bind dsRNA electrostatically, protect it from environmental factors and release it gradually on leaf surfaces and within tissues, thus extending the period of virus protection from a few days in the case of naked dsRNA to about 20 days in greenhouse tests, and offering virus protection against both insect-vectored viruses and fungal pathogens (Niño-Sánchez et al., 2022). DNA nano structures precisely engineered assemblies like tetrahedra and nanotube shave been employed to deliver siRNAs into gene silencing of the mature leaves of *Nicotiana benthamiana* and *Arabidopsis thaliana* without the need of external mechanical force (Zhang et al., 2019). Recently, LDH nanoparticles and other nano-carriers have been reported to deliver small RNAs deep into chloroplast-rich tissues and soft polymer nanoparticles of about ~20 nm have been proven to be able to pass through root cell walls and deposit in meristematic and vascular tissues (Gao et al., 2023; Yong et al., 2022) (**Figure 1**).

Several recent reviews have synthesized these innovations in plant nanotechnology. Cunningham et al. (2018) introduced a conceptual framework to explain biomolecule delivery by nanoparticles, which mainly focused on the ability of nanomaterials to penetrate cell walls and deliver DNA, RNA, and proteins to plants. Demirer et al. (2019) in their respective articles have presented nanomaterial-based gene delivery as a radically new technique for the genetic transformation of Agrobacterium and biolistics, highlighting the material classes and uptake routes. Review by Yong et al. (2022), Liu et al. (2023) and Gao et al., 2023), and others have elaborated on the design strategies of nanoparticle carriers, breakthroughs in nano-enabled CRISPR delivery, and the expanding horizon of plant nanobiotechnology for sensing, fertilization, and stress alleviation (Gao et al., 2023; Wang et al., 2023; Yong et al., 2022). Together, these publications indicate that nanomaterials are capable of (i) passing through plant barriers in a gentler and more extensive manner than traditional methods, (ii) safeguarding and gradually releasing delicate cargos like RNA, and (iii) being chemically adjusted for targeted tissues and organelles.

From the point of view of plant breeding and agricultural deployment, understanding what is missing could be summed up as the absence of a review that not only examines methods and materials but also raises much harder, trait-related questions. To date, the articles focusing on nanotechnology have hardly ever considered the nano-delivery platforms in relation to the specific breeding targets such as yield components, plant architecture, disease resistance genes, or stress tolerance alleles. Besides, they do not intuitively think about how nanoparticle-enabled delivery can be harmonized with the contemporary breeding pipelines, speed breeding, and regulatory frameworks for transgene-free edited crops (Cunningham et al., 2018; Demirer et al., 2019). On the other hand, the majority of plant-breeding-oriented CRISPR reviews consider delivery as a common limitation, making only a brief mention of nanotechnology (Chen et al., 2024; Xie et al., 2022). Consequently, there exists a conceptual gap between the capabilities of nanocarriers in plants and the requirements of breeders to be able to use genome editing as a means for crop improvement. To contextualize the shift from conventional transformation to nanocarrier-mediated delivery, key differences are summarized below (**Table 1**).

**Table 1:** Key contrasting features of conventional transformation and nanocarrier-mediated delivery.

Feature	Conventional methods (agrobacterium, biolistics)	Nano-enabled delivery
DNA integration	Often random integration	Potentially DNA-free
Host range	Genotype-dependent	Broader (in principle)
Tissue requirement	Tissue culture-dependent	Potentially tissue culture-free
Cargo type	Primarily DNA	DNA, RNA, RNPs
Transformation efficiency	Moderate–high (optimized systems)	Variable, often lower
Regeneration	Required	Often unresolved bottleneck
Regulatory perception	GMO (in many jurisdictions)	Potentially non-GMO (context-dependent)
Scalability	Established	Emerging

### 1.4 Scope and objectives of the review

This review is intended to bridge that gap by positioning nanotechnology not merely as a fancy gene-delivery method but as a future platform for nanoparticle-enabled precision breeding. Firstly, on plants as the central theme, we sketch the major classes of nanocarriers compatible with plants along with the parameters of their design and functionalization that determine their interaction with the tissues of the plant (Section 2). Secondly, we analyze the mechanisms of uptake, transport, and subcellular trafficking of nanoparticles in relation to plant anatomy and physiology (Section 3). Thirdly, we combine the current achievements in genome-editing payload delivery to cells via nanoparticles including DNA, RNA, and RNP complexes and also explicate what has and what has not been accomplished in terms of stable, heritable edits (Section 4). Finally, we scrutinize the ways in which these platforms can be utilized to meet the specific breeding goals of yield and plant architecture, disease resistance, abiotic stress tolerance, and RNA-based crop protection, also noting the realistic applications in the near term as well as the over-hyped prospects (Section 5) (Chen et al., 2024; Ni et al., 2021; Nivya et al., 2023; Vats et al., 2024; Xie et al., 2022; Zeng et al., 2020). After that, we consider the environmental fate, biosafety, regulatory classification, and socio-economic factors associated with nano-enabled genome engineering in crops (Section 7). We then move on to the future of smart, stimulus-responsive nanocarriers as well as their coming together with synthetic biology and advanced breeding strategies (Section 8) (Gao et al., 2023; Raza et al., 2023; Wang et al., 2023; Yadav et al., 2023).

So, as a whole, we posit the idea that nanoparticle based delivery, when it is a conscious development with a deep understanding of plant physiology and breeding situations, has the potential to fundamentally change the way genome editing is used. It can become not so much a highly specialised, genotype restricted means, but rather a more standard, easily accessible tool that can be taken to the field for transgene-free crop improvement. The main point here is not if nanoparticles are capable of penetrating plants they obviously are but rather if they could be used, in a trustworthy and safe manner, to provide the exact molecular edits that are required by modern agriculture (Wang et al., 2023). For a clear understanding, the studies discussed about has been interpreted based on their demonstrated level of biological validation: (i) nanoparticle uptake, (ii) transient expression or silencing, (iii) detectable genome editing activity, (iv) regeneration of edited plants, and (v) heritable transmission.

## 2 Design principles of nanoparticles for plant systems

### 2.1 Fundamental design considerations governing biological fate

Nanocarrier design in plant systems should therefore be evaluated using criteria that reflect plant-specific constraints. Key considerations include cell wall traversal, surface retention, tissue specificity, controlled cargo release, reproducibility, and phytotoxicity. In contrast, mammalian-derived metrics such as renal clearance or the enhanced permeability and retention (EPR) effect are not directly applicable. The *in vivo* movement of nanoparticles encounters numerous challenges, referred to as “biological barrier”, while their behaviour relies on a combination of different physicochemical characteristics (Yu et al., 2026).

#### 2.1.1 Size, charge, and morphology

Size of the carriers is crucial in regulating biodistribution. Particles smaller than ~5-10 nm undergo rapid renal clearance, while those larger than ~200 nm are prone to sequestration by the spleen and phagocytosis by macrophages in the liver (the mononuclear phagocyte system, MPS) (De Jong and Borm, 2008). The “sweet spot” for systemic delivery, particularly for exploiting the Enhanced Permeability and Retention (EPR) effect in tumours, is generally considered to be between 10 and 200 nm, allowing for prolonged circulation and passive accumulation.

Surface charge is reported to affect the adsorption of protein, intracellular transport, and toxicity, which is measure as zeta potential (Weiss et al., 2021). Bhattacharjee (2016) discussed the electrostatic interactions with negatively charged membranes encouraged by positive charge surfaces that supports cellular uptake but can also induce cytotoxicity and adsorption of nonspecific protein “opsonin” thus leading to prompt removal by MPS. Contrarily, particles with neutral or mildly negative charge (-10 to +10 mV) are responsible for lower protein adsorption, extended movement, and constant biodistribution in cells.

#### 2.1.2 Surface chemistry and functionalization

Surface chemistry is the final layer of control, determining “stealth” properties and active targeting. Polyethylene glycol (PEG) conjugation, or PEGylation, remains the gold standard for creating a hydrophilic steric barrier that minimizes protein adsorption and MPS recognition (a property known as “stealth”) (Suk et al., 2016). However, immune responses against PEG (anti-PEG antibodies) have prompted exploration of alternatives like zwitterionic polymers or polysarcosine (Chen et al., 2023). For active targeting, surface functionalization with ligands—antibodies, peptides, aptamers, or small molecules—enables receptor-mediated endocytosis in specific cell types. A key challenge is the “binding-site barrier,” where overly dense ligand presentation can hinder deep tissue penetration (Rosenblum et al., 2018; Rosenblum et al., 2020). This has spurred the development of “smart” systems where targeting ligands are shielded during circulation and exposed only at the disease site.

#### 2.1.3 Cellular uptake and transport pathways

Roots and leaves are the primary sources of nanoparticles uptake in the plants. They enter in plant cells through stomata, cuticle openings, and epidermal cells in roots (Hong et al., 2021). These particles then adopt apoplastic or symplastic routes inside xylem and phloem and facilitate systemic distribution between roots and shoots (Avellan et al., 2022; Rani et al., 2023; Talebi, 2024). Size, surface charge, and plant species are the main determinants of their translocation in plants (Huang et al., 2022a).

#### 2.1.4 Biocompatibility, toxicity, and degradation

Biocompatibility of nanocarriers in various biochemical mechanisms is dependant on their size andsurface characteristics (Sanità et al., 2020). Low concentrations of these particles reportedly improve plant growth, whereas high concentrations may cause disruption in nutrient absorption, oxidative stress, and affect cell organelles (Ali et al., 2021; Ali et al., 2022; An et al., 2022). Generation of reactive oxygen species and membrane lipid peroxidation are responsible for toxicity issues that significantly affect plant growth. Biodegradation of nanoparticles is an intricate phenomenon because surface alterations and environmental interactions can compromise their bioavailability and toxicity, highlighting the need for careful nanoparticles design to ensure safe agricultural utilization (Islam, 2025).

#### 2.1.5 Targeting efficiency and tissue specificity

The development of efficient nanoparticles emphasizes on tissue-specific delivery and improved targeting efficiency (Nasr et al., 2026), which can be achieved by modifying nanoparticle size and surface charge properties to enable targeted cellular entry. Small sized particles can easily penetrate cell wall barriers and intracellular pores, whereas surface functionalization alleviates off-target localizations and other phytotoxicity risks (Rajesh, 2025; Gautam et al., 2025).

## 3 Major classes of nanoparticles and their biomedical/agricultural relevance

Advancements in nanoparticle engineering have introduced new possibilities to understand and manipulate biological systems at molecular and cellular levels. By manipulating matter at the nanoscale (typically 1-200 nm), scientists can create vehicles with precisely tuned physicochemical properties that interact with biological environments in unprecedented ways (Mitchell et al., 2021). This tunability defines the prospects of nanomedicine, as nanocarriers can bypass biological hurdles if strategically engineered with controlled design and surface modifications, thus optimize therapeutic outcomes and minimize off-target effects (Anselmo and Mitragotri, 2021; Deshmukh et al., 2024). A broad spectrum of nanocarrier systems has been described in the literature. However, their validation in plant systems remains uneven. While many studies report uptake or short-term activity under controlled conditions, relatively few demonstrate consistent and reproducible genome editing in whole plants. Consequently, nanoparticle classes must be assessed not only by their composition but also by the robustness and reproducibility of plant-specific evidence. The nanoparticle classes reviewed below are not equivalent in plant systems. Among the available nanocarrier platforms, carbon nanotubes, carbon dots, and layered double hydroxides provide the most consistent evidence for plant uptake and transient cargo delivery. DNA nanostructures introduce a high degree of programmability, although their application in plants remains largely exploratory. Lipid- and polymer-based carriers offer considerable flexibility and scalability but still lack robust validation at the crop level, while mesoporous silica systems, despite their high loading capacity, are less established for reproducible in planta editing. Accordingly, the following sections assess each class not only in terms of composition but also with respect to plant-specific evidence, reproducibility, and known limitations.

### 3.1 Lipid nanoparticles

#### 3.1.1 Structure and delivery advantages

Lipid nanoparticles (LNPs) have emerged as one of the most successful non-viral delivery platforms, spectacularly validated by their use in mRNA COVID-19 vaccines (Hou et al., 2021). Typically composed of ionizable lipids, phospholipids, cholesterol, and PEG-lipids, LNPs excel at encapsulating and protecting nucleic acids (mRNA, siRNA) from degradation.

#### 3.1.2 Nucleic acid delivery and gene editing applications

The endosomal escape capability of LNPs, often facilitated by the ionizable lipid’s protonation in acidic endosomes, is crucial for delivering cargo to the cytoplasm (Schoenmaker et al., 2021). Recent studies show that how lipids are organised inside a cell (for example, phase separation) largely determines the rate at which nucleic acids are released and the therapeutic efficacy, particularly challenging targets like neurons cells (Liu et al., 2023). Advances in production, particularly microfluidic mixing, have made it possible to produce highly reproducible batches with better encapsulation efficiency and narrow size distribution.

### 3.2 Polymeric nanoparticles

#### 3.2.1 Natural vs synthetic polymers

PNPs are usually made from either natural or synthetic polymers, both of which provide several advantages in delivering genetic materials and administering different agrochemicals (Sidhu et al., 2025). Since natural polymers are biodegradable, it is much safer and environmentally friendly to use them in various kinds of plant applications. Commonly used natural polymers include alginate, chitosan, cellulose, and starch (Zhou et al., 2025). A study mentioned the use of chitosan nanocarriers in chilli and tomato for improving nutrient uptake and enabling efficient delivery of agrochemicals or RNA molecules with reduced environmental risks (Ullah et al., 2026). Contrarily, synthetic polymers are proven to exhibit better structural integrity with advanced degradation phenomenon, thus facilitating controlled transport of biomolecules. Biodegradable polymers like poly (lactic-co-glycolic acid) (PLGA) and polyethylene glycol (PEG) copolymers form the backbone of this versatile class (Lu et al., 2023).

#### 3.2.2 Controlled release and targeting

Polymeric nanoparticles, due to their extraordinary control over drug release profiles, ensure continuous release over weeks to stimuli-responsive bursts triggered by pH, enzymes, or redox potential. They can comfortably be altered for targeting, and possess biocompatible degradation products (Wang et al., 2025b). Recent innovations emphasize on complexing nucleic acids with cationic or charge-altering polymers to create polyplexes, with microfluidic methods improving homogeneity and transfection efficiency (Zhou et al., 2022; Yousefi Adlsadabad et al., 2024).

### 3.3 Mesoporous silica nanoparticles

#### 3.3.1 High loading capacity

MSNs possess a high surface area and tunable, uniform pore structures that can be loaded with substantial therapeutic payloads (Kankala et al., 2020), with rigid inorganic framework that offers stability, while the silica surface is readily functionalized with various organic groups (Watermann and Brieger, 2017).

#### 3.3.2 Stimuli-responsive release

The dual framework of MSNs allow for gated drug release systems responsive to specific triggers like low pH in the tumour microenvironment (Luo et al., 2024; Hasan et al., 2024). Ongoing research aims to optimize pore architecture, enhance biodegradability, and reduce potential long-term toxicity to facilitate clinical adoption.

### 3.4 Carbon-based nanoparticles

#### 3.4.1 Carbon nanotubes

Carbon nanotubes are commonly used for biosensing and thermal therapy due to their tremendous mechanical potential and electrical conductivity (Murjani et al., 2022). Nevertheless, they face significant challenges due to their hydrophobicity and toxicity. Hence, improving aqueous dispersion and biocompatibility of carbon nanotubes require complex modifications in their surfaces with covalent oxidation or non-covalent coating with surfactants or polymers (Khan and Alamry, 2022). Functionalized CNTs are an extensive delivery system for various compounds and genes as they possess needle-like structures supporting membrane penetration (Zare et al., 2021).

#### 3.4.2 Carbon dots and graphene derivatives

Carbon dots (CDs) and graphene derivatives such as quantum graphene derivatives (GQDs) are carbon-based nanocarriers characterized by their ultra small size (approx.10 nm), more surface area and better surface integrity. These traits make them highly suitable for effective intracellular transport of biomolecules (DNA/RNA) in plant cells. Stable nanocomplexes are formed by combining carbon dots with plasmid molecules that remain protected from enzymatic degradation. Studies have demonstrated the significant role of carbon dots-based particles in successfully delivery of DNA into various crops like wheat, rice, and mung bean (Wang et al., 2022a). Additionally, the applications of carbon dots as low toxic and multifunctional nanocarriers are reported for improving soil health and stress tolerance in plants (Andleeb et al., 2025). Similarly, quantum graphene derivatives also possess the same biological traits, including robust intracellular penetration, nanoscale size, and advanced surface functionalization, making them an efficient tool for transporting double-stranded RNA or other biomolecules across plant cell wall, as investigated in wheat for delivery of dsRNA (Gyawali et al., 2024).

### 3.5 Layered double hydroxides

#### 3.5.1 Clay nanosheets for nucleic acid delivery

LDHs are anionic clays consisting of positively charged brucite-like layers with intercalated anions and water molecules. This layered structure allows for high loading of anionic drugs or biomolecules (e.g., DNA, siRNA) through ion exchange (Rodríguez et al., 2024). Their degradation in mild acidic conditions makes them suitable for pH-responsive release in tumour tissues. Coatings can further modulate their release profile and enhance colloidal stability.

#### 3.5.2 Stability and slow-release properties

Being positively charged metal hydroxide layers, LDHs nanocarriers are structurally stable and possess regulated release properties. They are protected in different layers, thus preventing them from enzymatic degradation in soil or plants. Furthermore, they release active compounds which are readily available to plants. For instance, slow release of dsRNA on plant leaves by LDH-based BioClay systems was reported, that ensure viral protection in tobacco plants for prolonged period of time (Hernandez, 2021). Likewise, phosphate-loaded LDHs were demonstrated as slow-release fertilizers for extended availability of nutrients to tomato and maize plants (Hassanzadeh et al., 2024).

### 3.6 DNA/RNA origami and programmable nanostructures

#### 3.6.1 Self-assembled nucleic acid nanostructures

Moving beyond traditional materials, nucleic acid origami utilizes the predictable base-pairing of DNA or RNA to construct arbitrarily shaped 2D and 3D nanostructures with nanometer precision (Liu et al., 2018; Madsen and Gothelf, 2019).

#### 3.6.2 Precision targeting and programmability

These structures are inherently biocompatible and can be programmed to display multiple targeting ligands with exact spatial orientation, enabling ultra-specific targeting and multivalent binding (Jiang et al., 2021). Although nuclease stability and scalable production are still the major fields of research, they represent a frontier of “smart, programmable” nanomedicine.

Unlike polymeric or inorganic nanocarriers, the synthesis of DNA and RNA origami is not only highly programmable and precise but also very expensive and low throughput. Consequently, their use mostly remains limited to controlled laboratory studies and target validation. Large-scale crop improvement or field applications of DNA and RNA origami are not economically feasible at the moment.

### 3.7 Recent advances in DNA nanostructures

#### 3.7.1 Controlled release mechanisms

Programmable dynamic DNA devices can release cargo when exposed to specific molecular cues. For example, a biomimetic “nano-Actinia” system consists of deformable DNA strands fixed to a gold nanoparticle core, and when irradiation to light, the DNA strands change their shape and release the encapsulated hydrophobic drugs (Jiang et al., 2023). Similarly, DNA dendrimers that are engineered to respond to the high levels of glutathione in cancer cells can lead to the simultaneous delivery of chemotherapy drugs, thereby reducing harm to healthy tissues and showing the possibility of spatiotemporal drug delivery approaches.

#### 3.7.2 Programmable membrane interaction

The most transformative aspect however might be that DNA nanostructures can directly engage and remodel lipid bilayers - a function very closely related to living cells. Researchers have developed dynamic DNA origami structures which, once bound to vesicle membranes and triggered by specific DNA “fuel” strands, confine controlled curvature changes, mimicking the action of proteins like clathrin or dynamin (Liu et al., 2022). A pioneer study reported that DNA shells anchored with cholesterol could be used to program membrane budding. Such a capability is a critical step towards preparation of synthetic cellular components or advanced delivery vesicles.

#### 3.7.2 Enhanced stability

The major drawback of DNA nanostructures is their sensitivity to nuclease degradation (Lacroix and Sleiman, 2021). This problem is being solved by various strategies such as assembling the structures in physiologically relevant monovalent ion solutions (e.g. Na+) or using chemically modified nucleotides (e.g. phosphorothioates) which significantly enhance the stability of the structures in biological fluids and consequently make the *in vivo* applications more feasible.

Nanoparticle design is categorized along a trend of increasing sophistication and convergence. While traditional material platforms such as LNPs and polymeric NPs are being fine-tuned through advanced manufacturing and computational modelling, Simultaneously, emerging platforms like DNA origami are bringing unprecedented levels of programmability and functionality. The future will be characterized by the creation of hybrid and multimodal systems that integrate the strengths of multiple materials, for instance, a lipid-coated DNA origami structure or a polymeric core decorated with targeting aptamers and contrast agents.

## 4 Nanoparticle-mediated genome editing

Importantly, the outcomes of delivery reported in the literature are highly context-dependent. Protoplast-based assays, transient tissue systems, and whole-plant transformations are different, and each introduces different biological constraints. In this section, studies are very clearly categorized based on whether they show uptake, transient expression, editing activity, regeneration, or heritable transmission. Although the design of nanocarriers has been rapidly progressing, the majority of the reported achievements are still limited to protoplast systems, transient assays, or controlled environments. Demonstration of stable, heritable genome editing in intact crop plants are still limited, highlighting a big gap between experimental feasibility and translational deployment. For instance, nanotechnology offers new avenues for bringing innovation and improvement in the production of crops, thus encouraging novel strategies in genome editing and plant biotechnology. Recently, Nanoparticle-mediated genome editing is being adopted in plants at an early stage (Lv et al., 2020). The scope of plant nanobiotechnology is purely dependant on the methods of delivering nanoparticles. Several studies have been conducted on the uptake of nanoparticles, but the transport mechanism of nanoparticles in plants should be well understood to design their delivery methods. Similarly, it is imperative to modulate physical properties of nanoparticles to address physiology and genome editing purposes in plants.

### 4.1 CRISPR/Cas delivery formats

Nanoparticle mediated delivery has clearly expanded the toolbox for plant genome engineering, but the literature remains uneven across systems and endpoints. In most cases, the strongest evidence is for cargo uptake, transient expression, or RNA silencing, whereas stable, heritable editing in elite crops remains uncommon. The distinction between these outcomes is central to interpreting the field accurately. CRISPR/Cas system face considerable challenges in its efficient delivery to target cells or tissues, with current delivery methods including viral vectors, non-viral vectors, and physical means of delivery. Among them, viral vectors are widely used as they integrate CRISPR/Cas9 coding sequences into viral genome, thus release ultimate gene complex into the infected cells/tissues. Nonetheless, there are higher risks of mutations and limited delivery of CRISPR/Cas9 DNA associated with this method (Duan et al., 2021).

The nanomaterial-based gene delivery system has already been adopted in mammals, specifically exploited commercially for incorporating genetic material into human cells. Despite of the rising interest towards nanobiotechnology for their substantial role in plant engineering, only fewer studies focused on the nanoparticles-mediated delivery of nucleic acids into the plant cells (Arshad et al., 2025). Yet, the nanoparticles have been exploited for their accurate and precise gene delivery into plants. Nano-particles are being widely used in in nanobiology and genetic engineering because of their specific small size, expanded surface area, and low toxicity. They contribute significantly in genetic material and protein transmission in plant cells. In agriculture, these materials can be readily used to enhance the production techniques of plants by improving target specific delivery of biomolecules such as nucleic acids and proteins.

#### 4.1.1 Ribonucleoproteins, DNA-based, and mRNA-based delivery

Different ways of targeting genes in the plant cells through CRISPR/Cas9 include encoding Cas9 protein and sgRNA into the same plasmid vector, delivering mRNA of Cas9 protein and sgRNA, and dispersed delivery of Cas9 protein and sgRNA, or delivering the ribonucleoprotein complexes (RNP). The most direct method is the delivery of RNP which ease the gene editing process without having the need of expression and transcription, thus minimize the chances of off-targets and toxicity. However, the efficient delivery of RNP faces significant difficulties, therefore, making it challenging to be effectively delivered into plant cells or nucleus without an appropriate vector (Zhang et al., 2021). Cas9 protein components, being unstable and of large molecular weight, require an efficient vital nano-delivery method (Lee et al., 2025).

Nanoparticle-mediated delivery systems encourage the transport of varied CRISPR delivery formats such as ribonucleoproteins (RNPs), messenger RNA (mRNA), and DNA in plant cells (Tashima, 2025). Multiple nanoparticle-mediated delivery systems have been reported in plants such as exosome-like nanoparticle, lipid complexes, cationic polymers and gold-based nanoparticles (Wang et al., 2025b).

Multiple factors responsible for efficient delivery of ribonucleoproteins (RNPs) in plants mainly include particular plant specie, type of tissues (such as protoplasts), delivery method, quality of RNP, selection of nuclease, and gRNA sequence (Zhang et al., 2021). Nanoparticle-mediated delivery systems, in essence, paved a new way for promising and efficient delivery of RNP molecules into plant cells. Among them, lipid nanoparticles (LNPs) are being widely used for RNP complexes for gene editing in higher plants (Onuma et al., 2023). They are biodegradable and biocompatible, while protecting genome-editing systems in cells. Mahmoud and Dutt (2024) reported that a lipofectamine-mediated transfection for DNA-free genome editing of citrus protoplast cells using a Cas9/gRNA RNP complex resulted in the production of transgene free genome edited citrus plants. Additionally, gold nanoparticles have also emerged as versatile tools for CRISPR/Cas9 RNP delivery. The absorption of RNPs on the surface of nanoparticles without enzymatic degradation has been made possible by combining AuNPs with cationic polymers (Pourali et al., 2023). These nanocarriers optimize electrostatic interactions while encouraging precise genome-editing with less toxicity. To accurately assess the practical utility of these delivery innovations, it is essential to distinguish between cellular uptake and genuine breeding relevance. Research in this field is characterized by varying biological endpoints: (1) internal cellular uptake, (2) transient expression or silencing, (3) detectable editing activity, (4) regeneration of viable plantlets, and (5) confirmed heritable transmission. For these nanoparticle-mediated systems to be considered a definitive advancement in crop improvement, the evidence must extend beyond protoplast or transient assays.

### 4.2 Nanoparticle-mediated delivery of DNA

In plants, DNA/RNA mediated gene delivery is being frequently practiced (Lv et al., 2020). Nanoparticles composed of DNA are efficient, non-toxic, and easy to metabolize as being basic components of genetic material in cells of all living organisms. Besides, they have considerable stability and prevent biodegradation by nucleases (Chandrasekaran, 2021). Among them, carbon carriers, metallic (goldnanosphere, goldnanorods, and iron oxide clusters) and silicon-carriers, and biological carriers (liposomes) are commonly used in plants (Dhondiyal et al., 2025).

#### 4.2.1 Carbon dots and carbon nanotubes-mediated DNA delivery

*Carbon dots, (carbon-based microscopic nanoparticles)*, are reportedly used as genetic cargo in plants with their suitability of bypassing the cell wall. They efficiently transmit different exogenous nucleic acid components such as DNA (She et al., 2025a) and siRNA (Delgado-Martín et al., 2022) to engineer plant genomes. As most of the metal-based nanoparticles are of environmental concerns, the degradation of carbon dots into CO_2_ and H_2_O makes them more environmentally friendly to be applied for plants, thus contributing to sustainable agricultural ecosystem (Li et al., 2023). She et al. (2025b) developed an efficient modified carbon dot (MCD)-mediated transformation system for successfully delivering DNA plasmids into wheat seeds. Cas9 mutation efficiency was evaluated by using seed based transient transformation by combining MCD with CRISPR vectors targeting *NLR1* and *NLR2* genes. The transferred DNA plasmid was detected in all regenerated seedlings, depicting efficient transformation. Hence, this system has great potential for application in other crops to improve their agronomic characteristics.

Carbon nanotubes (CNTs) have been widely acknowledged as a medium to get through the plant cell wall (Pawar et al., 2023). This capability is mainly credited to their high aspect ratio and needle-like figure which helps their alignment and penetration through the cellulose-hemicellulose network, rather than pore formation by the nanotubes. Some researchers also think that temporary nanoscale disruption or local changes of the wall polymers might take place during the entry; still, stable pore formation in plant systems has not been reported so far. Therefore, CNT-mediated entry should be predominantly regarded as a shape-facilitated passive penetration method as opposed to a biologically induced pore formation mechanism (Demirer et al., 2019).

#### 4.2.2 Gold nanomaterial-mediated DNA delivery

Among metallic nanoparticles, gold nanoparticles are of special concern with respect to their manipulative size, quick synthesis, and comfortable surface alteration. They are widely used in delivering biomolecules like DNA and peptides in plants and animal cells. After *Agrobacterium*-mediated transformation, gold nanocarriers (AuNPs) delivery is the most promising tool in plant transformation to carry exogenous biomolecules into plant cells by crossing the cell wall, with more DNA delivery efficiency (Yan et al., 2022). AuNPs are being increasingly adopted in engineering animal cells with limited cytotoxic effect, however scanty studies are present emphasizing their roles in plants. Nevertheless, various sizes and shapes of AuNPs are used to ensure promising delivery of DNA-Cy3 into plant cells via injection method (Zhi et al., 2022). Additionally, mesoporous silica particles (MSNs) are used in combination with gold nanocarriers for co-delivering DNA and proteins into the white epidermal cells in onion.

### 4.3 Nanoparticle-mediated delivery of RNA

#### 4.3.1 Lipid and polymeric nanoparticle-mediated delivery of mRNA

Among non-viral vectors, lipid nanoparticles (LNP) and polymeric nanoparticles are widely used in effectively delivering mRNA into plant cells. Though, due to prolonged stability and storage hurdles associated with LNPs, development of efficient mRNA delivery vectors with robust transfection ability was substantial. For this reason, polymer-base nanoparticles have gained more interest due to their diverse structure, streamlined function, and uniform stability (Piotrowski-Daspit et al., 2020). Polymeric nanocarriers used for mRNA delivery include polyester, polyethyleneimine, poly amino acids, and dendrimers (Huang et al., 2022b). Polyethyleneimine (PEI) are involved in mRNA complexation by providing more positive charges due to the presence of several amino groups. They have been successfully employed in *invitro* delivery of mRNA. Besides, there are other polymers-based mRNA nanocarriers like chitosan, poly (diethylaminoethyl methacrylate) (PDMAEMA), poly(glycoamidoamine) (PGAA), and zwitterionic phospholipidated polymers (ZPPs) (Huang et al., 2022b). The scope of Lipid and Polymeric Nanoparticle-mediated delivery of mRNA is more in clinical studies as compared to plants.

#### 4.3.2 Exosome-like nanoparticle delivery of mRNA, sRNA, and miRNA

Transmission of nucleic acids such as mRNA, sRNA, and miRNA, proteins and lipids are executed effectively by exosome-like nanoparticles due to their smaller size; they are reported to increase the functional stability of their contents, thus ensuring the availability of bioactive molecules (Dad et al., 2021). Exosomes are composed of lipids, proteins, and nuclear reagents which function a nanocarriers in both plant and animal cells. Recently, they have been found in transferring RNA molecules from plants to pathogens and pests, thus minimizing their virulence. For instance, in plant-fungi interactions, host cells of *Arabidopsis* deliver sRNA into the fungal cells of *Botrytis cineraria* through extracellular vesicles like exosomes, which supports the notion of using such vesicles in delivering sRNA to silence virulence of pathogens and promote resistance (Cai et al., 2018).

#### 4.3.3 LDH-mediated delivery of siRNA and dsRNA

Exogenous siRNAs and double-stranded RNAs (dsRNAs) are reportedly involved in silencing the gene expression of viruses, fungi, or other pathogens for their protection. Nevertheless, when sprayed on leaves of a plant, they are unstable with a high risk of degradation and easily washed away. Interestingly, Layered double hydroxide (LDH) clay nanosheets are not linked with this issue and are non-taxic and difficult to be washed (Lv et al., 2020). Remarkably, dsRNA can easily be absorbed by these nanocarriers and up taken by the plants while silencing the target viral DNA to escape infection. Despite of being highly efficient in dsRNA delivery, the real mechanism of dsRNA uptake by the plants and interaction between plants and pathogenic pests need further experimental studies. To facilitate comparison across material classes, their key properties and limitations are summarized in **Table 2**.

**Table 2:** Comparative overview of nanocarrier systems for plant delivery.

Nanocarrier type	Primary cargo	Delivery route	Key advantage	Major limitation
Lipid nanoparticles	mRNA, RNPs	Foliar, infiltration	Biocompatibility	Stability in plants
Polymeric NPs	DNA, RNA	Root, foliar	Tunable properties	Variable efficiency
Mesoporous silica	DNA, proteins	Injection	High loading capacity	Limited plant validation
Carbon nanotubes	DNA, RNA	Foliar	Cell wall penetration	Potential toxicity
Carbon dots	siRNA, DNA	Foliar	Small size, fluorescence	Limited scalability data
LDH nanosheets	dsRNA	Foliar spray	Sustained release	Limited editing evidence
DNA nanostructures	DNA, RNA	Infiltration	Programmability	Cost and scalability

### 4.4 Advantages of transgene-free editing

The production of genetically modified organisms (GMOs) is coupled with substantial regulatory and biosafety challenges, which has led to the increasing concern for transgene-free editing approaches. The integration of Cas9 and foreign DNA plasmids in traditional CRISPR/Cas systems limit their acceptability at commercial level (Chuang et al., 2021). Therefore, adoption of non-transgenic approaches for crop improvement is imperative, while facilitating extensive application of genome editing strategies. Nanoparticles enable genome editing without using any traditional transformation methods (e.g. *Agrobacterium*-mediated transformation) and without the delivery of DNA plasmid, as they contain Cas9/gRNA complexes or mRNA/gRNA, making them most reliable tools for the production of GMOs. Co-delivery of dsDNA with nanoparticles appear to be integrated in the desired genome, thus bypassing the hurdles in delivering editing moieties. Furthermore, the integration of RNA as a template with nanoparticles or any viral vectors can also alleviate the issues associated with transgene-free editing (Tsanova et al., 2021). It was reported that direct uptake of nanoparticles such as carbon nanotubes, metal or metal oxide NPs, and quantum dots by the plants indicates their ability produce transgene-free crops. Among them, gold nanoparticles have been reported to possess physical and chemical stability coupled with exceptional phyto-compatibility (Vats et al., 2022).

#### 4.4.1 Avoidance of somaclonal variation

Somaclonal variation include genetic changes that occur during dedifferentiation (callus formation) and regeneration phases through *invitro* plant tissue culture. Majority of the nanoparticle-mediated systems are dependant on plant tissue culture techniques for regeneration of transgenic plant. Interestingly, nanoparticle-mediated gene delivery has the potential of avoiding or shortening different phases of plant regeneration through tissue culture, thus avoiding somaclonal variation. Due to their direct delivery of genetic material in to the plant cells, nanocarriers-based edited cells are able to develop into whole plants naturally, consequently circumventing prolonged callus induction and regeneration phases and escaping somaclonal variation (Quiroz et al., 2025). Following this principle, nanocarriers are potentially used to deliver gene-editing reagents to all kinds of plant cells, including meristematic cells, which leads to the generation of chimerically gene edited plants, without dedifferentiation phase (Hu and Liu, 2025).

Furthermore, increasing emphasis is being given placed on introducing tissue-culture free approaches. One of such approaches is pollen magnetofection that enable direct gene transfer into viable pollen, thus encouraging uniform development of transgenic plants any surpassing the limitations of conventional strategies. The first successful application of this method was reported in lily through staining for β-glucuronidase (GUS). Zhang and his coworkers established a lily pollen transfection system on a large scale with more effective transfection (Zhang et al., 2023).

#### 4.4.2 Reduced regulatory constraints

Nanoparticle-mediated genome editing clearly dominates the conventional transgenic approaches in terms of reduced regulatory constraints. Production of transgene-free plants by this novel technique itself can overcome most of such regulatory frameworks. Different types of nanocarriers such as CNTs or biopolymer-based materials can efficiently deliver CRISPR/Cas complexes or other genetic cargoes into plant cells without integrating with foreign DNA, which explicitly ensures the generation of genome edited plants that, in certain jurisdictions, may not be classified as GMOs (Das et al., 2026).

#### 4.4.3 Improved public acceptance

Public acceptance is crucial in widespread adoption of novel technologies. Nanotechnology-based genome editing is associated with the targeted modifications without integration of foreign DNA, thus mitigating major public concerns about conventional transgenic plants. It can further foster more acceptance among consumers and industry stakeholders, specifically related to safety, environmental and ethical concerns (Mobeen et al., 2025). Additionally, public engagements on agricultural nanotechnology highlights the benefits, for example, limited use of agrochemicals and the promotion of sustainable agriculture, thus fostering greater acceptance when supported by transparent regulatory frameworks and appropriate safety evaluations (Murikipudi et al., 2026).

### 4.5 Prospects for organelle genome editing

#### 4.5.1 Chloroplast and mitochondrial targeting

Plant cells are composed of cell wall and chloroplasts along with a larger central vacuole; they all are surrounded by a cell wall that offers protection against osmotic stress. Besides, nucleus, mitochondria and chloroplasts represent the chief targets for genetic transformation and exogenous delivery of DNA. Transmission of genetic material has been reported multiple times in microbial and animal cells through various techniques such as electroporation, sonoporation, chemical treatment, microinjection, and nanotechnology. Nevertheless, they have not been implemented within plan cells with much success owing to distinct cellular and biological barriers. Moreover, organelle genome editing was rarely documented prior to advances in nanoparticle-mediated delivery approaches.

There are a variety of nanomaterials that deliver different cargoes in plants, but understanding their entry pathways into plant cells needs further attention. In general, lipid exchange envelop penetration (LEEP) has been demonstrated for entry of nanotubes into the cell wall of plant cells (Law et al., 2023). Likewise different gold nano particles like nanorods have also been proposed which do not need any physical entry to deliver their cargo. The delivery of DNA and RNA into specific organelles in plant cells by using polymer conjugated carbon nanotubes has been reported (Demirer et al., 2019; Kour et al., 2024; Kwak et al., 2019).

Nanomaterials which have been used for delivering plasmid DNA into the nucleus include mesoporous silica, carbon dots, carbon nanotubes, and peptide-based nanocomplexes. They prevent the DNA cargo degradation by inserting cationic polymers to form electrostatic complexes with DNA. Peptide-based nanoparticles were used by Miyamoto et al. (2018) to deliver nucleic acid contents into the nuclei of different plants. Similarly, they reported the delivery of exogenous DNA into the mitochondria of Arabidopsis leaves by combining mitochondria-targeting (Cytox) and cell penetrating peptides (CPPs), which can be passed through the plasma membrane in a variety of plant cells. Moreover, the delivery efficiencies of CNTs in targeting mitochondria have been shown to increase by 30-fold in comparison with the peptide only methods (Das Karmakar et al., 2025). The principal technique for delivering genetic material into the chloroplasts was particle bombardment (Ozyigit and Yucebilgili Kurtoglu, 2020) which was unable to target specific organelles with low transformation efficiency. To circumvent this, the utilization of nanoparticles was introduced for targeted delivery of DNA to chloroplast without using specialized equipment and laborious dealing with protoplasts. Among all, SWCNTs and peptide-based nanocomplexes are specifically used to incorporate foreign DNA in to chloroplasts. Santana et al., 2022 reported the use of carbon-based nanomaterials for targeted delivery of plasmid DNA to chloroplasts and investigated their impact on plant cell. They synthesized carbon dots (CDs) for chloroplasts targeted chemical delivery, and single-walled carbon nanotubes (SWCNTs) for gene delivery in Arabidopsis plants. In another study, polyethylenimine-coated single-walled carbon nanotubes (PEI-SWCNT) were investigated to deliver foreign DNA to algal (Chlamydomonas) chloroplasts, coupled with examining SWCNT uptake affecting by algae cell wall barrier, followed by SWCNT’s effect on chloroplast photosynthesis. The results indicated that highly charged PEI25k-SWCNT exhibited higher potential for DNA delivery into algal chloroplasts (Newkirk et al., 2023). Additionally, stable plastid transformation was reported in tobacco and rice by using peptide-mediated DNA delivery system in combination with Spectinomycin/Streptomycin (Sp/Str) selection, resulted in successful transmission of integrated exogeneous genes into the next generations of all plants (Odahara et al., 2022).

#### 4.5.2 Challenges and emerging nanocarrier solutions

Despite of the prospects for specifically targeting plant organs, there are numerous biological challenges that hinder the efficiency and broad-spectrum adoption of organelle genome editing in plants. Among them, the rigidity of the plant cell wall, plasma membrane, and the double-membrane structures around organelles are the major obstacles in free successful transport of the delivery cargoes such as DNA, RNA, or RNPs (Kour et al., 2024; Kwak et al., 2019). Biologically, genome editing systems are required to address multiple copies of the organelles like mitochondria and chloroplasts to attain homoplasmy, posing a considerable challenge (Abe and Numata, 2025; Wang et al., 2025a).

Rapid advancements in nanotechnology have introduced innovative solutions to mitigate these challenges, specifically the nanocarriers like carbon nanotubes, metal-based nanocarriers, and mesoporous silica nanocarriers have exhibited a robust potential for efficient delivery of nucleic acids and genome-editing molecules in plant cells. They are capable of entering into the plant cell by easing delivery of reagents as well as acting as barrier to avoid their degradation. MSN are reported to facilitate loading and stable release of genetic cargo, thus enabling specific organelle genome editing, whereas peptide-based nanoparticles are used to transport nucleic acids into various plant organelles, such as chloroplasts or mitochondria, through organelle-specific targeting (Law et al., 2023; Yong et al., 2024). Nevertheless, some other obstacles, such as nanoparticle toxicity, broad-scale application, and precise organelle targeting require special focus to ensure vast adoption of nanoparticle-based organelle genome editing in plant biotechnology. A major limitation across studies is the lack of standardized metrics for delivery efficiency and editing frequency. Reported efficiencies vary widely depending on species, tissue type, and experimental conditions, and reproducibility across laboratories remains insufficiently assessed. Furthermore, scalability is constrained by challenges in nanoparticle synthesis, delivery uniformity, and plant regeneration capacity.

## 5 Applications in crop improvement

**Figure 2:**
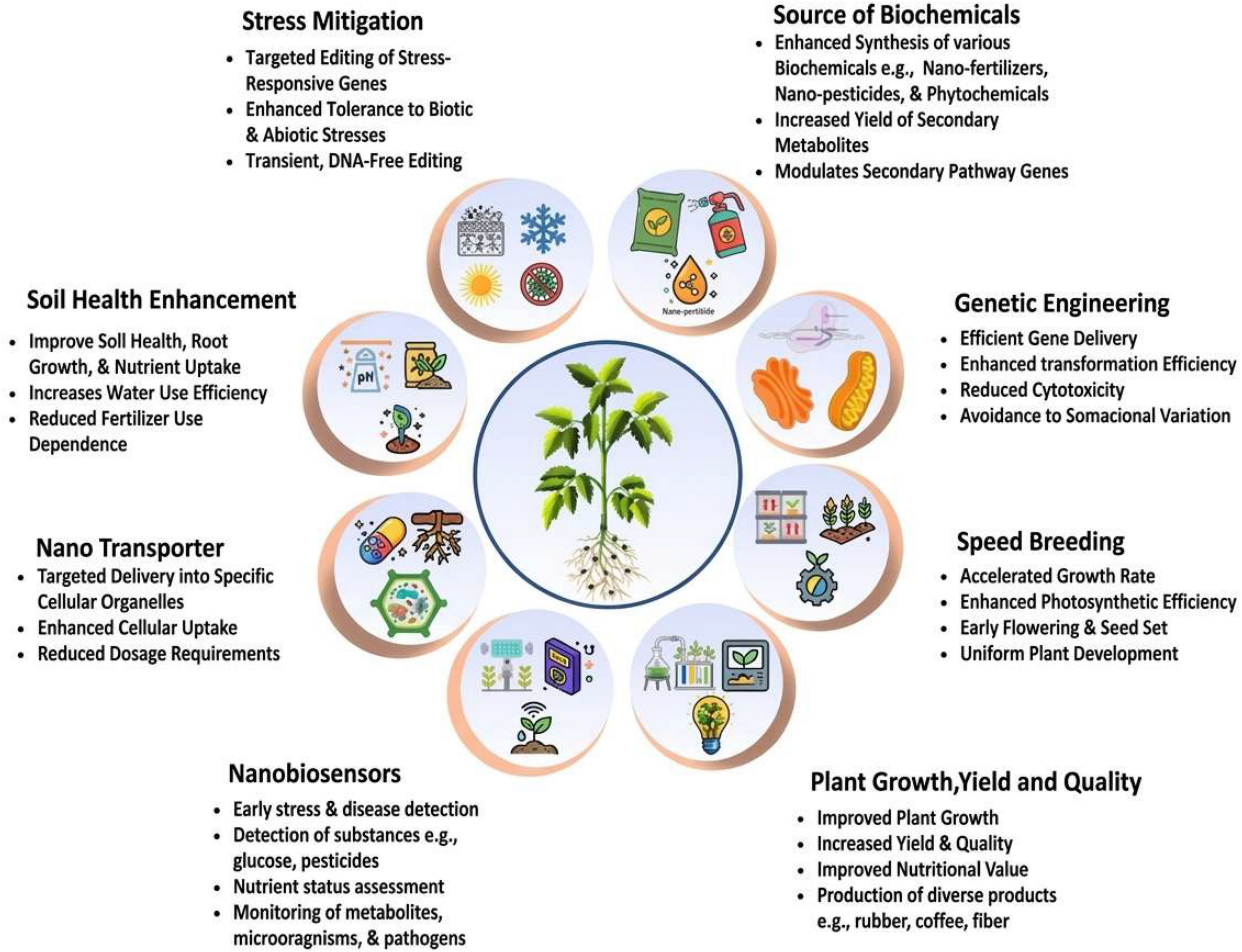
Applications of nanoparticle-mediated genome editing in plants for enhanced breeding.

This section distinguishes three levels of evidence: proof of delivery, proof of target perturbation, and proof of breeding value. For yield and architecture traits, the present literature mainly supports transient target testing in mature tissues or protoplasts, not stable, heritable nano-delivered edits in elite crops. By contrast, disease resistance loci such as MLO and SWEET are better supported by conventional editing literature and therefore provide the most defensible near term targets for nano-enabled trait validation rather than immediate field deployment. Where no field-relevant nano-delivered editing example exists, that absence is stated explicitly. Nanocarrier platforms are only complex just to solve real problems that are necessary for breeding. For crops, that would be three things: (i) the ability to directly edit or modulate high-value loci in elite, often transformation-recalcitrant germplasm, (ii) rapidly target discovery and validation under authentic stress and disease conditions, and (iii) the provision of deployable RNA-based crop protection tools that can be used together with genetic resistance (Chen et al., 2024; Nivya et al., 2023). Currently, almost all robust plant nanotechnology evidence that have been transient expression and RNAi; stable, heritable edits via nano-delivery in crops are still lacking (Cunningham et al., 2018; Zhang et al., 2019). Hence, this section considers “applications” as a deliberate strict separation between what has actually been demonstrated and what is realistically the next (**Figure 2**).

### 5.1 Trait targets for precision editing

#### 5.1.1 Yield improvement and plant architecture

The example of rice loci demonstrates how precise genetic edits can dramatically change the production of the harvest. Gn1a/OsCKX2 is responsible for a cytokinin oxidase-dehydrogenase; when the enzyme activity is lowered, the amount of cytokinin in the inflorescence meristems highly increases, and thus the number of spikelets and grain yield go up, while a few alleles also strengthen the culm and develop the crown root. DEP1, a non-typical G-protein γ subunit, influences panicle compactness and erectness and thus canopy architecture and light interception (Zhao et al., 2016). CRISPR edits on Gn1a and DEP1 in combination with elite backgrounds have led to the production of high-yielding, erect-panicle rice lines by the means of Agrobacterium or biolistics. At present, top-class mega-varieties are usually not at the same level as the most advanced gene-edited prototypes due to the fact that transformation and regeneration strongly depend on genotype, are slow, and are often accompanied by undesirable somaclonal variation (Chen et al., 2024; Nivya et al., 2023). In the case of globally vital but transformation-recalcitrant mega-varieties, particularly indica and hybrid rices, as well as wheat and maize, the use of such edited alleles is still a matter of long backcrossing or complex transformation optimization. This is exactly the point where nano-delivery might have a pivotal role in the game. It brings the option of editing yield loci right in the case of elite cultivars thereby bypassing the need for highly transformable proxies.

**Figure 3:**
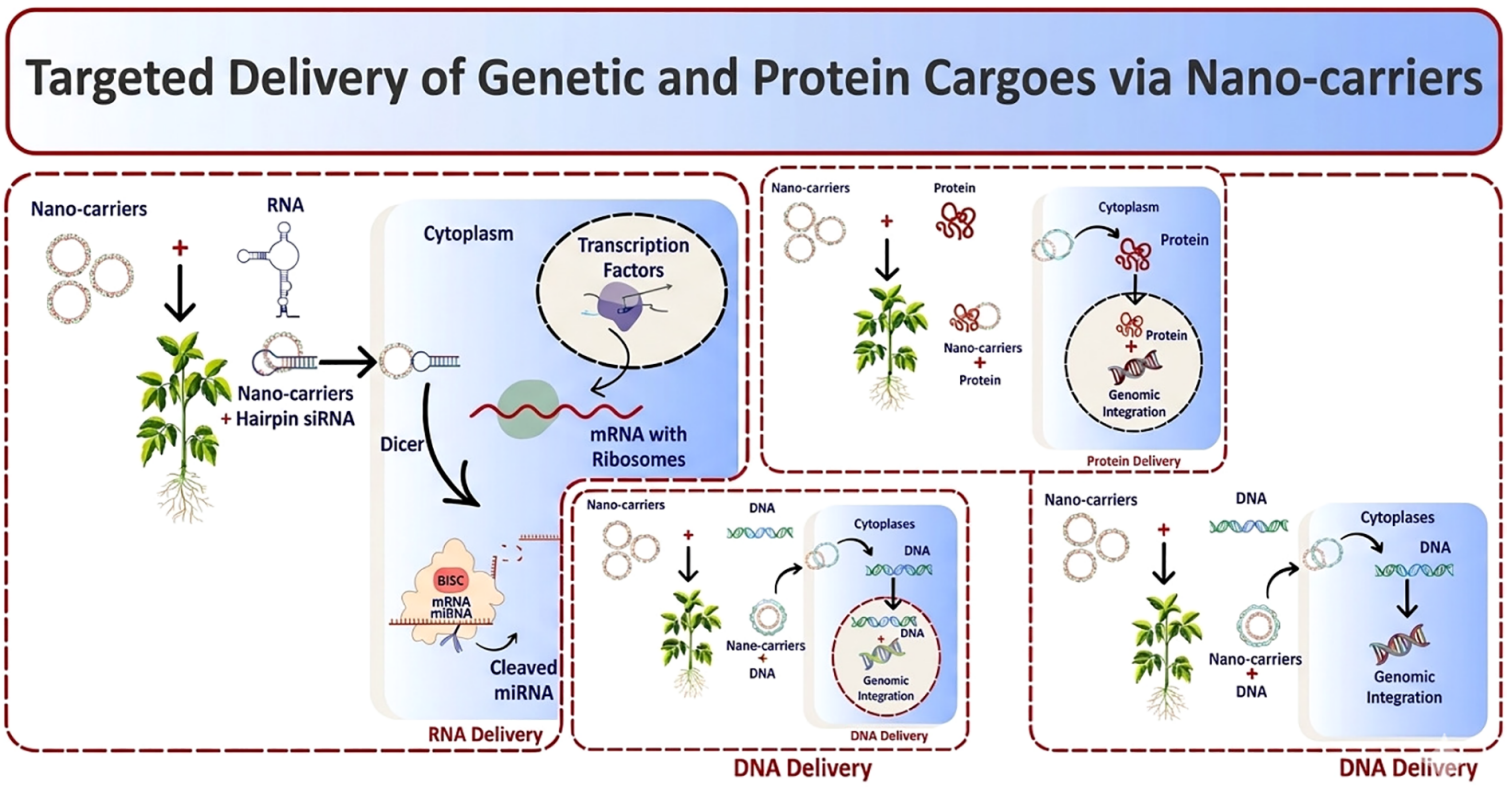
Targeted delivery of genetic & protein cargoes via nano-carriers.

Presently, plant nanotech offers a few relevant delivery vehicles. One of these is carbon nanotubes (CNTs) which are high-aspect-ratio and polymer functionalized. They have been found to be capable of delivering plasmid DNA into the leaf structure of arugula, wheat, and cotton, thus leading to a transient green fluorescence protein (GFP) expression, with only slight damage to the cells and what is very important no detectable genetic recombination of the plasmid DNA (Demirer et al., 2019). Besides, Cy3-tagged DNA–CNT conjugates can be detected in mesophyll cells by means of fluorescence, thus proving that the nanocarriers get through the cell wall and plasma membrane of mature tissues (Demirer et al., 2019). By means of mesoporous silica nanoparticles (MSNs) with pores of about 3 nm, plasmid DNA and small molecular weight agents have been successfully introduced into isolated plant cells and leaves of maize. The release of cargo is done by gold nanoparticle “caps” which are removed under the action of the specific stimuli (Cunningham et al., 2018). Such platforms supply an unambiguous template for translocating CRISPR plasmids, mRNA, or even RNPs into reproductive meristems or targeted cereals & developing panicles (**Figure 3**).

Not a single study so far has been able to demonstrate that stable, heritable edits at yield loci via nanoparticle delivery in a major crop, and therefore this point needs to be made very clear. The most nano-mediated gene delivery leads to transient expression, and at best, somatic modification in mature leaves (Cunningham et al., 2018; Zhang et al., 2019). On the other hand, the mechanisms for yield editing nanoparticles being taken up by meristematic tissues, getting to germline precursors, and unloading genome editing cargos without persistent integration are, in fact, in agreement with the demonstrated functions of soft polymer nanoparticles and high-aspect-ratio nanomaterials which travel through vasculature and are found in growing tissues (Gao et al., 2023; Yong et al., 2022). In the case of an affordable near-to-midterm scenario, nanoparticle methods will almost certainly be the first ones to be employed for in planta screening and trait validation. For instance, the transient expression or knocking down of *Gn1a*, *DEP1*, or other panicle regulators in certain tissues of the elite lines to evaluate phenotypic changes before making permanent edits by any transformation way that is currently reliable in that crop. After achieving strong meristem or germline targeting, one can then use these same platforms to direct editing. These systems have been used for rapid phenotype testing and target validation, but not as an evidence that nano-enabled breeding is already field ready.

#### 5.1.2 Disease resistance via susceptibility genes (MLO, SWEET and related targets)

On the genetics side of disease resistance, the case is already quite convincing. The *MLO* locus in barley and other organisms exemplifies the kind of resistance that lasts long and covers a wide range of diseases: the recessive mlo alleles have ensured the effective control of powdery mildew in European barley cultivars for over 30 years, which is a remarkably long time when compared to the lifespan of race-specific R-genes. In a similar fashion, mlo-based strategies are being used in the research of wheat, tomato, and grapevine (Jacott et al., 2021; Dreiseitl, 2025; Li and Panstruga, 2025; Dreiseitl, 2025). One of the most common diseases in rice is bacterial leaf blight caused by the bacterium *Xanthomonas oryzae* pv. *oryzae* that through transcription activator-like effectors (*TALEs*) hijacks specific *OsSWEET* sugar transporter genes to turn them into susceptibility factors. By CRISPR editing the TALE-binding elements in the promoters of *OsSWEET11*, *OsSWEET13*, and *OsSWEET14*, rice lines with broad-spectrum bacterial blight resistance were generated that also lack any measurable yield penalties, even in commercial elite cultivars (Zeng et al., 2020; Ni et al., 2021; Duy et al., 2021; Zeng et al., 2020). Until now, most of these improvements have been done in traditional ways. Nanotechnology has two possible roles to play here:

##### 5.1.2.1 Trait discovery and rapid allele testing

By the use of nanocarriers, the application of siRNAs, antisense oligos or transient CRISPR components into mature leaves or panicles of target cultivars can be the way of loss-of-function mimic of candidate susceptibility genes (e.g. MLO homologues, SWEET or other transporters) under infection conditions. Thereby, breeders and pathologists would have the opportunity to check if knocking out a certain gene leads to the desired resistance in their genetic backgrounds thus, saving them time and money by not having to produce stable edited lines.

##### 5.1.2.2 Direct editing in elite backgrounds

On the condition that nanocarrier systems are designed suitably, base editors or prime editors as RNP complexes can be delivered to developing floral tissues or seeds, thus creating allelic series at susceptibility loci in one single generation of elite lines. These alleles could have varied precise patterns of promoter mutations or amino acid substitutions thus, enabling the adjustment of resistance vs. agronomic penalties.

The idea of the first one is partly supported by existing data. In one experiment Demirer et al. (2019) found that single-walled carbon nanotubes (SWNTs) were capable of delivering siRNAs into the leaves of *Nicotiana benthamiana* thus, achieving >70% knockdown of an endogenous gene with strong and reproducible phenotypes. The researchers claim that the effectiveness of siRNAs delivered through the internalization of DNA nanostructures into mature leaves depends on the size, stiffness of the nanostructure, and the siRNA attachment sites as well. In *N. benthamiana* leaves tetrahedral DNA nanostructures and nanotubes internalize and deliver siRNAs to silence a constitutively expressed GFP transgene, with the efficiency depending on nanostructure size, stiffness and the location of siRNA attachment sites (Zhang et al., 2019). Layered double hydroxide (LDH) nanoparticles of about 40 nm have been proven to quickly enter *N. benthamian*a leaves and deliver siRNAs that downregulate target transcripts by more than 70% within one day of application (Yong et al., 2022). These platforms are the perfect tools for the rapid, non-integrative target validation that is lacking in many CRISPR breeding programmes at present.

On the other hand, the idea of second-level heritable edits at S-genes through nanoparticle delivery is still a dream for scientists working on field-relevant crops. Plant transformation by means of nanoparticles has been a popular topic in recent reviews; however, most of the reviews pinpoint the same issue: while nanocarriers are proven to be efficient in delivering DNA, RNA, and proteins into plant cells, the transition from transient expression to stable germline editing is still only a conceptual idea and lacks substantial experimental work (Cunningham et al., 2018; Demirer et al., 2019). Hence, a serious use of nanoparticle-facilitated editing at *MLO* or *SWEET* loci would not only call for better germline targeting but also detailed off-target investigations, including the way the nanomaterials themselves behave in plant tissues. At present, these systems have been employed as the best tools for rapid phenotype testing and target validation, not as evidence that nano-enabled breeding is already field ready.

#### 5.1.3 Abiotic stress tolerance and resource-use efficiency

##### 5.1.3.1 Drought, salinity, temperature resilience

Abiotic stress tolerance, which includes drought, salinity, heat, and nutrient-use efficiency, is an area where the physiology and polygenic inheritance of the plant intersect. Rice, Wheat and Maize editing projects have been focused on the different components of the plant cell, such as transcription factors (e.g. DREB, NAC, bZIP), signalling kinases, ion transporters (e.g. HKT, NHX), aquaporins, and regulators of root system architecture. CRISPR-based changes in these genes can raise the stress tolerance levels significantly under controlled conditions but it is quite a challenge to bring this technology down to the farm as the field trials results vary with different genotypes and it is difficult to combine several tolerance mechanisms in one genotype (Chen et al., 2024; Raza et al., 2023; Xie et al., 2022).

There are two ways in which nanotechnology can interact with this field. To begin with, a number of nanofertilizers and nano-biostimulants, such as MSNs, metal oxide nanoparticles and carbon-based materials, have been reported for enhancing nutrient uptake, photosynthetic performance, and reactive oxygen species scavenging in a variety of crops (Sadati et al., 2022; Manzoor et al., 2022; Raza et al., 2023), though the results are highly dependent on the context and are sometimes inconsistent (Manzoor et al., 2022; Raza et al., 2023; Yadav et al., 2023). Secondly, and more importantly, nanocarriers can be used to introduce the editing reagents or regulatory RNAs into the stressed parts of the plant to help confirm the physiological effects of priority targets (e.g. transiently silencing a negative regulator of drought tolerance to see the physiological effects), allow the spatial imposition of edits (e.g. root- vs. shoot-specific modulation), and probably creating edited lines in stress-adapted local cultivars that are hard to transform. For instance, clay nanoparticles and MSNs have been utilized for the delivery of the protective or regulatory molecules to roots and shoots, thus leading to the changed reactions to salinity and heavy metal stress, while LDH-based platforms are being developed to deliver the dsRNAs that target the fungal virulence genes in rice sheath blight (Manzoor et al., 2022; Raza et al., 2023). These experiments are still mostly at the proof-of-concept level and do not yet involve genome editing, but they show that nanoparticles can directly interact with the stress physiology of mature plants.

Therefore, in the short term, target validation through nanoparticle-enabled editing would be the most feasible contribution in this area. This would involve the use of nano-delivered siRNA or transient CRISPR systems for perturbing stress-related genes in mature plants under realistic stress regimes, thus determining which loci are worth being permanently edited through whichever transformation method is currently stable in that crop.

### 5.2 Nanocarrier-assisted RNAi silencing for crop protection

#### 5.2.1 siRNA/dsRNA delivery for pest and pathogen control

Plant nanotechnology is mostly a matter of time before it comes to the field for the plant siRNA/dsRNA delivery area. The topical RNA sprays for viruses, insects and fungi that are controlled by them are degraded rapidly by UV, rain and nucleases; thus naked dsRNA is usually only detectable on leaves for a few days. A high-pressure spraying of naked dsRNA on *N. benthamiana* with a GFP transgene did not result in GFP silencing, and the small RNA profiling showed that siRNAs formation was very low, thereby indicating that the uptake barrier was the main problem (Qiao et al., 2021; Zhang et al., 2024a).

Layered double hydroxide (LDH) “BioClay” nanosheets actually change this being the case. LDH nanosheets to which dsRNA is electrostatically bound remain stable on plant surfaces for a long time i.e. from days to weeks, and the dsRNA is slowly released as the clay dissolves. It was found that one foliar application of LDH–dsRNA could provide virus protection for 20 days in both cowpea and *N. benthamiana*, while that of naked dsRNA only for a few days (Ray et al., 2026). Work carried out afterwards found that BioClay formulations not only can protect the plants against various viruses but they may also be able to diminish insect vectors’ egg-laying and survival (Niño-Sánchez et al., 2022). Therefore, clay nanoparticles are changing the persistence and effectiveness of exogenous RNAi in plants entirely.

Studies reported recently have extended the applications of LDH-based delivery to siRNA and thus different host–pathogen systems as well. Yong and co-authors demonstrated that nanoparticles of LDH about 40 nm size can very quickly enter not only the leaf cells but also the chloroplasts that are intact and effectively provide the siRNAs that reduce the endogenous transcripts by more than 70% in *N. benthamiana.* The fluorescent labelling also shows that a lot of nanoparticle accumulates in the chloroplast-rich tissues (Yong et al., 2022). Carbon dot preparations with an approximate size of 5–10 nm have been utilized for the insertion of siRNAs into *N. benthamiana* via a straightforward spraying, thereby resulting in strong visible phenotypes and target mRNA as well as protein levels being significantly decreased. In addition, the smallness and hydrophilicity of carbon dots render them easy passage through the cuticle and cell wall without any detectable toxicity (Schwartz et al., 2020). DNA nano structures tetrahedra, nanotubes, and other architected assemblies are the next step in control, as siRNAs can be attached to certain parts of the scaffold to regulate their accessibility and efficiency of silencing (Zhang et al., 2019).

From the perspective of crop improvement, the mentioned technologies could be implemented in three interconnected ways:

- Direct crop protection. Nanocarrier-protected dsRNAs targeting viral, fungal, or insect genes can be sprayed on crops to provide temporary, sequence-specific protection. On the one hand, experiments on tomato and chickpea demonstrated that BioClay-like substances could extend the protection against Botrytis cinerea. On the other hand, LDH–dsRNA sprays have been able to lessen virus transmission by aphids in model systems (Niño-Sánchez et al., 2022). Presently, large-scale trials are underway to evaluate the effectiveness under real environmental conditions (Raza et al., 2023).
- Host gene modulation and target validation. The use of transient silencing of host genes classical susceptibility genes, negative regulators of defence or stress tolerance to assess resistance-growth trade-offs in elite germplasm is one of the possible applications. Given that siRNA/dsRNA have a temporary effect, one can perform such tests to obtain results without passing through the stage of GM plants creation, and later permanently editing the positive targets.
- Functional genomics and tissue-specific phenotyping. The change in nanoparticle chemistry and the application (foliar vs. root vs. injection) may allow delivery bias towards certain tissues or developmental stages. Through this, gene function can be disrupted in a more localized or time-limited manner than the whole-plant mutants, thus enabling the discovery of gene functions hidden by pleiotropy or developmental compensation.

#### 5.2.2 Spray-induced gene silencing (SIGS)

Spray-induced gene silencing (SIGS) represents an emerging RNAi-mediated approach that supports plant protection and functional genomics by serving as transgene-free and environmentally friendly substitute to traditional plant disease control methods. Compared with host-induced gene silencing (HIGS), SIGS involves the foliar application of double-stranded RNA (dsRNA) or small interfering RNA (siRNA) onto plant surfaces, followed by their uptake by plant tissues and/or invading pathogens, where it activates precise gene silencing through RNA interference pathway (Chen et al., 2025). This technique harnesses RNA interference to attain targeted gene silencing in insects/pests, fungi, or viruses without inducing permanent genomic changes in plants, which is why it is widely accepted as a better alternative to HIGS that cause genomic modifications (Chen et al., 2025; Parise et al., 2024). In comparison with traditional RNA sprays, nanoparticle-formulated SIGS systems typically not only exhibit improved adhesion to the leaf cuticle but also resist environmental degradation more effectively. The use of layered double hydroxides and polymeric-based carrier materials, for example, can increase the retention time by protecting the RNA against UV degradation and water removal; however, quantitative comparison across different crops and formulations are remain limited (Ray et al., 2026).

Previous reports pinpointed the ability of SIGS to suppress disease incidence in plants by controlling genes of fungal pathogens (Wu et al., 2024). While traditional pesticides or agrochemicals leave their residues in the ecosystem and affect beneficial organisms, SIGS is biodegradable and thus mitigates risks to environment and human health. Due to its sequence specificity, dsRNA in SIGS facilitates targeted silencing of pathogen genes without affecting beneficial organisms such as insects or soil microbes, thereby confirming its suitability for integrated pest management. Moreover, the studies verify its biodegradability prior to crops consumption by the consumers, addressing public concerns about pesticide residues and supporting compliance with food safety regulations (Christiaens et al., 2020).

Exogenous application of dsRNA is recognized by Dice-like enzymes into siRNAs that are linked to RNA-induced silencing complex (RISC) (Das and Sherif, 2020; Venu et al., 2024). The complex is then directed to mRNA by the resulting siRNA, consequently leading to its natural degradation and gene silencing in target organism (Chen et al., 2025). SIGS has been extensively applied against several phytopathogens. Nevertheless, the uptake potential differs among several pathogens. Efficient uptake of dsRNA is reported in different fungal pathogens, such as *Botrytis cinerea*, *Sclerotinia sclerotiorum*, *Rhizoctonia solani*, *Aspergillus niger* and *Verticillium dahlia* (Ouyang et al., 2025; Gebremichael et al., 2021). Similarly, effective absorption of dsRNA was reported in both *Fusarium oxysporum* and tissues of tomato plant (Ouyang et al., 2023). Conversely, no uptake of dsRNA was reported in *Colletotrichum gloeosporioides*, while weak uptake in the beneficial fungus *Trichoderma virens* (Ray et al., 2022). With ongoing innovations, SIGS is likely to play a pivotal role in climate-smart agriculture by reducing pesticides dependency, and facilitating sequence-specific targeted control of invasive plant pathogens.

#### 5.2.3 Field-level applications

The nano-RNAi technology is much nearer to market introduction than the nanoparticle-assisted genome editing. A number of regulatory bodies in different regions worldwide have considered dsRNA-based products as biopesticides rather than GM crops. Besides, the employment of biodegradable, non-toxic nanocarriers like LDH and some carbon-based materials may additionally facilitate the removal of regulatory obstacles (Raza et al., 2023). The main implication for breeders and crop protection specialists in the short term is that nano-enabled RNA sprays will most probably be commercially available years before nano-enabled CRISPR edits, thus, the two technologies should be used strategically like genetic resistance through edited S-genes and stress loci over the long term, together with rapidly reconfigurable, nano-protected RNA sprays which can be updated as pathogen populations evolve.

### 5.3 Synergies between plant biochemistry, physiology and nanotechnology

#### 5.3.1 Nanoparticle–plant metabolic interactions

The efficiency and specificity of nanoparticle-enabled trait modulation are dependent on plant physiology and biochemistry to be almost equal as on nanocarrier design. The composition of a leaf cuticle, the density and behaviour of stomata, the pH of apoplast, the redox status, the porosity of cell wall and the composition of xylem and phloem sap all determine nanoparticle adhesion, penetration, transport and cargo release (Gao et al., 2023; Wang et al., 2023). Despite this, numerous plant nanotechnology studies that consider plants as inert substrates, still apply a “one-size-fits-all” formulation across species and tissues without a detailed mechanistic investigation.

#### 5.3.2 Modulation of signalling pathways

In the case of traits of yield and architecture, to perform an effective editing or modulation, the target must be meristematic tissues and the reproductive organs in the development stage, where the decisions about tiller number, panicle branching and spikelet initiation are made. The vascular connectivity and hormone gradients of these tissues are very different from those in mature leaves. By designing nanocarriers that take advantage of certain transport routes by using ligands that attach to receptors that are enriched in inflorescence meristems, or by adjusting size and surface charge to be more compatible with phloem loading delivery efficiency at sites of interest could be increased to a great extent (Cunningham et al., 2018; Gao et al., 2023).

In the case of disease resistance and RNAi-based pest control, the interaction between the nanoparticle charge, size and hydrophobicity and the microenvironment of leaf surfaces will determine the duration of dsRNA activity, the quantity of dsRNA that enters stomata or cuticular cracks, and finally how it is transported to distal tissues. The effects of LDH–dsRNA and carbon dot–siRNA formulations reveal that most of the differences in the silencing efficiency between species or cultivars can be explained by leaf surface properties and internal transport differences, the latter being the main reasons, not RNAi machinery (Niño-Sánchez et al., 2022; Schwartz et al., 2020). Consequently, combining high-resolution imaging (confocal microscopy, TEM, X-ray fluorescence) and tracer studies (e.g. ICP-MS for metal-based NPs) with plant physiological measurements is the only way to go beyond empirical formulation tweaking.

#### 5.3.3 Precision trait modulation

The synergy that matters for breeding is the possibility of designing nanocarriers whose behaviour is predictable from plant traits that breeders already understand and manipulate. For example, knowing that a drought-tolerant ideotype has narrow, waxy leaves with reduced stomatal conductance immediately raises questions about foliar uptake of particular nanoparticle classes; this might favour root-applied or seed-applied nano-delivery for certain traits. Conversely, cultivars bred for high transpiration efficiency might be more amenable to xylem-mediated nanoparticle transport, enabling more effective delivery of editing reagents or regulatory RNAs to distal tissues.

Finally, environmental and soil biogeochemistry cannot be ignored. LDH-based carriers degrade into benign products (Mg/Al hydroxides, carbonate), and some MSNs dissolve into orthosilicic acid, which can even be beneficial for certain crops, whereas other materials (e.g. some metal oxides, poorly degradable carbon nanomaterials) may accumulate and interact with soil microbiota in poorly understood ways (Wang et al., 2023; Yadav et al., 2023). A serious crop improvement programme using nanotechnology must therefore be co-designed by nanomaterials scientists, plant physiologists, breeders and soil ecologists, with equal attention paid to trait modulation, plant health and environmental fate. In other words, nanoparticles should not be treated as magic bullets that somehow “solve” delivery once and for all. They are another powerful set of tools whose success in crop improvement will depend entirely on how intelligently they are integrated with what we already know about plant biochemistry, physiology and breeding.

## 6 Integration with modern breeding strategies

**Figure 4:**
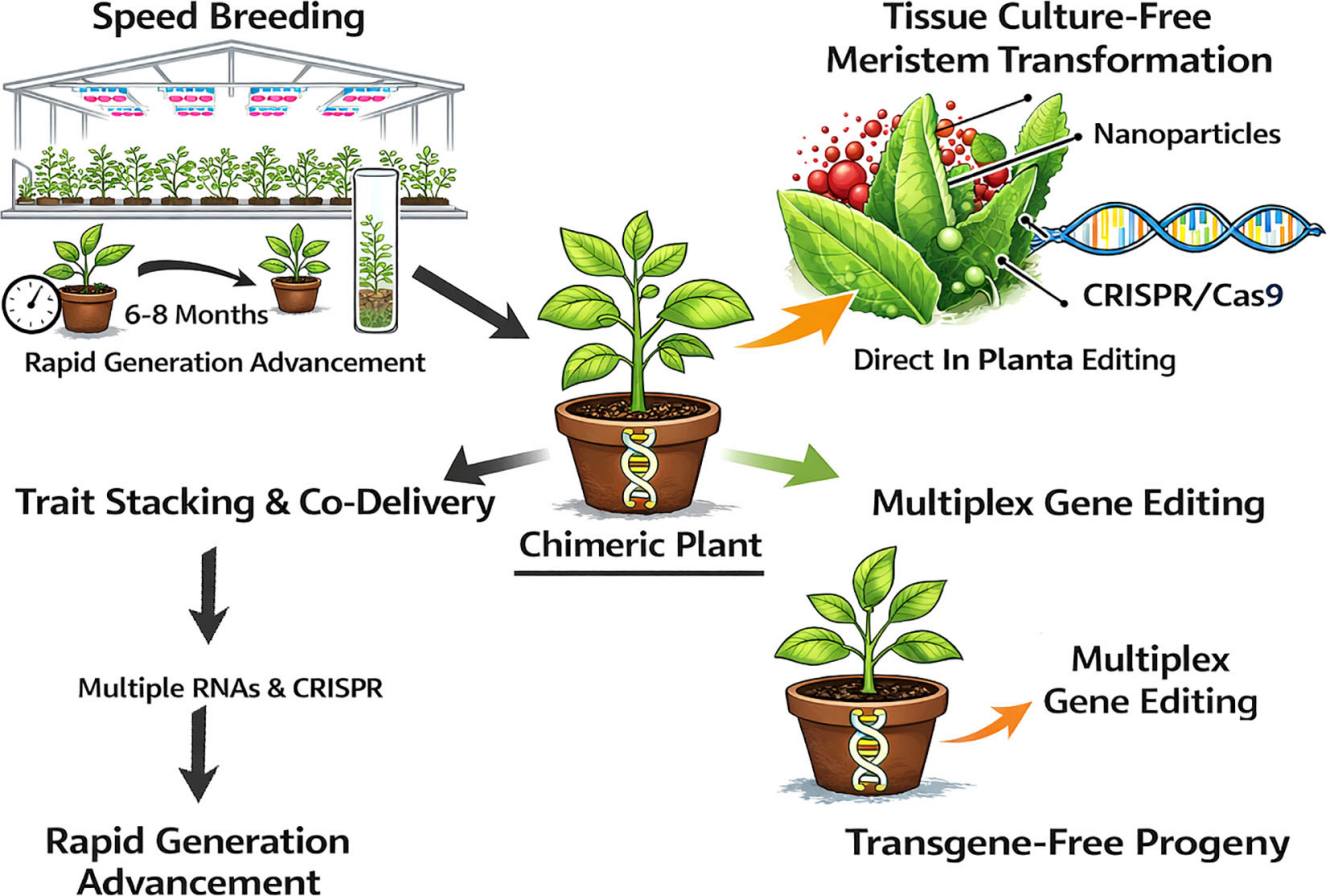
Integration of transgene-free editing with speed breeding and other modern breeding strategies.

A realistic workflow for nanodelivery in breeding begins with target nomination and cargo design, proceeds through transient validation in protoplasts or detached tissues, then moves to delivery in elite germplasm or meristematic tissue, regeneration and genotyping, phenotype confirmation under the relevant stress or disease condition, and finally fixation by selfing or backcrossing where needed. At each stage, the limiting factor is different: delivery efficiency, tissue specificity, regeneration, heritability, or field scalability (**Figure 4**).

### 6.1 Integration with speed breeding

Nano-based gene editing has paved a new way for transgenic approaches by using nanomaterials as gene carriers. Nevertheless, it is still challenging to get a speedy production of GM crops. This hurdle can be addressed by integrating nano-enabled gene editing with existing technologies. A speedy crop production can be accomplished using CRISPR/Cas systems by combining nanomaterials with speed breeding (Awan et al., 2024). Speed breeding involves the production of high-yielding and superior crops under controlled environmental conditions. It offers crop improvement at any stages of horticultural as well as agronomic crops in a controlled environment. Additionally, it also assists in producing *invitro* crops and nanomodified crops, ensures phytoremediation, improves plant’s resistance against various biotic and abiotic stresses, and integrates nanotechnology with the controlled ecosystem setup (**Figure 4**).

### 6.1 Rapid-cycle editing and trait validation

Combining speed breeding with nanoparticles can hasten the breeding cycle and optimize crop production (Raza et al., 2023). Delivery of genome-editing reagents can establish chimerically edited plants, which can then be subsequently advanced through multiple generations in a short time using speed breeding, thus facilitating the regeneration of transgene-free plants under optimized conditions (Ahmar et al., 2021).

Recently, nano osmics approach has been introduced to accelerate plant breeding, with its growing significance in modern crop improvement becoming progressively evident. Nano osmics is the interdisciplinary integration of nanotechnology with genomics in plant breeding which exploit plant’s specific traits at molecular level through nanocarriers and novel genomic approaches. Through this method, breeders can enhance plant traits such as yield, nutritional balance, disease resistance and stress tolerance precisely.

### 6.2 Meristem transformation and elite germplasm editing

#### 6.2.1 Tissue culture–free approaches

The delivery of genomic contents to meristematic zones of a plant leads to the generation of transformed plants with heritable features. Nanoparticles have the potential to bind with different biomolecules and encourage meristem transformation in shoot and root apices of a plant (Yan et al., 2023). Due to their very small size and unique surface properties, nanoparticle can easily enter into the cell wall through endocytosis, and then can comfortably move between cells, including meristem cells without damaging them. The successful delivery of DNA, RNA, or RNP complexes in the meristematic zones of a plant cell result in heritable changes in genetic makeup surpassing regeneration process through tissue culture techniques.

#### 6.2.2 Direct in planta editing

Nanoparticles take different routes to penetrate in meristem cells. One of those routes is passive diffusion that leverages natural ways to incorporate foreign DNA into the plant cell without using any external factor. Nanoparticles used for passive diffusion method mainly include Au, Zn, and carbon. Wang et al., 2022b tested the penetration of 5nm carbon dots in to the root tips of Rice Nipponbare plant by dipping them in a mixture of carbon dots and plasmid. The results confirmed the presence of nanoparticle-plasmid DNA complex which easily passed through the cell wall without any external aid. Penetration in shoot meristems can also be improved by incorporating nanocarriers with vacuum/syringe infiltration method. Syringe infiltration technique is applicable exclusively to leaf tissues (Shivashakarappa et al., 2025). Despite of being useful in DNA delivery, nanoparticles could be toxic to human health. Limited reports have mentioned the toxicity of zinc nanoparticles (ZnNPs) in different biological setups, including microorganisms, animals and human cells. Root tips of onion and faba beans are usually used to detect cytotoxicity and genotoxicity of nanomaterials and damage to DNA (Al-Saleh et al., 2025). Genotoxic effects of ZnNPs were investigated in the root tips of *Drimia indica* (Daphedar and Taranath, 2018). In current practice, the most realistic near-term role of nanodelivery is to accelerate early validation and reduce the number of lines that must enter conventional transformation or regeneration pipelines.

### 6.3 Trait Stacking via co-delivery systems

#### 6.3.1 Multiplex genome editing

Trait stacking, the insertion of multiple desirable traits into a single plant, is being used for crop improvement. Multiple agronomic traits like disease resistance, heat resistance, drought tolerance, are controlled by multiple genes which can be incorporated either by conventional or transgenic approaches in plants. Conventional approaches are time-consuming with hight chances of unpredictable gene expression, while transgenic approaches are coupled with regulatory, biosafety, and public acceptance fears.

#### 6.3.2 Multi-trait engineering

The nanoparticles-mediated co-delivery system is being used in plants for stacking a variety of traits which relies on the co-delivery of multiple genome reagents in single delivery pathway. With respect to CRISPR/Cas-based gene editing, it involves the co-delivery multiple sgRNA targeting several genes with Cas9 nuclease in the form of DNA, mRNA, or ribonucleoprotein (RNPs) complexes. Nanomaterials used for co-delivery applications in plants mainly include carbon-based nanocarriers, polymerous nanocarriers, mesoporous silica nanocarriers, and lipid-based nanocarriers (Ren et al., 2022; Purkait and Sontakke, 2023). Trait stacking using co-delivery of multiple editing reagents offer generation of transgene free and heritable edits in plants. RNPs delivered by nanoparticle-mediated delivery induce accurate genome edits in plant cells by avoiding off-targets, signifying the notion that this method is well suited for complex trait engineering (Zhang et al., 2021). In one study, co-delivery of nanoparticles was performed using DNA nanostructures and carbon nanotubes. Small interfering RNAs (siRNAs) were delivered into the leaves of tobacco by DNA nanostructures for effective gene silencing (Zhang et al., 2024b). Similarly, Odahara et al., 2021 reported that co-delivery of Cas9 RNP complexes carrying multiple gRNAs into the calli of *Arabidopsis* is possible by using cell-penetrating peptide (CPP)-modified Polyion Complex Vesicles (PICsome); the resultant mutations were detectable at the target sites. In another study, engineered dsRNA protein nanoparticles were employed to attain gene silencing in poplar and tobacco cells, thus confirming the co-delivery of RNA and protein cargoes in plant tissues (Sun et al., 2024). Collectively, the above-mentioned studies highlight the potential of nanoparticles to transport multiple biomolecular reagents into plant cells, thus proving themselves as promising tools for multiplex genome editing in trait stacking for crop improvement (**Table 3**).

**Table 3:** Nanocarrier platforms for crop-relevant trait targets: from yield and disease resistance to abiotic stress tolerance and RNA-based crop protection.

Trait class/breeding goal	Target gene(s)/process	Nanocarrier	Cargo	Species & tissue/route	Key outcome	TRL*
Yield & architecture – delivery in cereals	Reporter (GFP) as proxy for yield genes (*Gn1a*, *DEP1*)	Polymer-functionalized SWNTs	Plasmid DNA (~5.3 kb)	*N. benth.*, arugula, wheat, cotton; mature leaves, low-pressure infiltration	Strong transient GFP incl. wheat; ≈Agrobacterium; no DNA integration; low toxicity	Lab PoC (transient DNA delivery in cereals)
Yield & architecture – precise recombination	loxP cassette recombination (proxy for targeted edits)	Gold-capped MSNs	Cre recombinase (protein); plasmid DNA (earlier)	Maize (Lox-corn); embryogenic calli; biolistic	Cre delivered to maize cells; precise loxP recombination; up to ~30–40% recombined calli	Lab PoC (protein delivery, precise rearrangement in crop)
Disease resistance – S-gene concept (BLB in rice)*	*OsSWEET11/13/14* promoter EBEs	— (classical delivery; genetic context only)	CRISPR/Cas9, TALENs	Rice, multiple elite cultivars; callus-based	Edited EBEs give broad-spectrum BLB resistance; no yield penalty	Trait validated; nano-delivery not yet used
Virus resistance – crop protection	Viral coat proteins (e.g. CMV, PMMoV, BCMV)	LDH “BioClay” nanosheets	dsRNA	*N. benth.*, cowpea, tomato, chickpea; foliar spray	Single spray; protection ≥20 d; protects new leaves; aphid transmission blocked; naked dsRNA ≈5 d	GH + pre-field (virus control)
Fungal disease control	Fungal virulence genes (*Botrytis cinerea* targets)	LDH nanosheets (BioClay-type)	dsRNA	Tomato, chickpea; foliar spray	Reduced lesions vs naked dsRNA; extended protection window in GH assays	GH (fungal RNAi control)
Host gene modulation/S-gene validation	GFP, Mg-chelatase (proxy for *MLO*, *SWEET* etc.)	Carbon dots (~5–10 nm)	siRNA	*N. benth.*, tomato; foliar spray + surfactant	Strong local GFP silencing; chlorosis via Mg-chelatase knock-down; large mRNA/protein reduction; no wounding needed	Lab PoC (sprayable siRNA via cuticle)
Host gene modulation/S-gene validation	Endogenous transcripts in leaves	LDH NPs (<50 nm)	siRNA	*N. benth.*; foliar application	>70% mRNA knock-down in 24 h; strong chloroplast-rich cell uptake	Lab PoC (systemic siRNA delivery)
Abiotic stress – drought (biostimulant)	Stress pathways (antioxidants, drought-induced genes)	ZnO NPs	Zn nutrient	Wheat (and other cereals); seed priming/foliar	↑RWC, ↑antioxidant enzymes; 10–30% yield gain vs bulk Zn under drought (typical reports)	GH + some early field (agronomic tool)
Abiotic stress – salinity (biostimulant)	Ion homeostasis, salt injury mitigation	Nano-Si (SiO_2_ NPs)	Si (as H_4_SiO_4_)	Rice, wheat; root or foliar	↓Na^+^, ↑K^+^/Na^+^, improved growth, photosynthesis; sometimes yield benefits under salt	Pot/GH + limited field (agronomic tool)
Pest/disease control – RNA sprays	Viral, fungal, insect essential genes	LDH (BioClay), LDH NPs, carbon dots	dsRNA/siRNA	Model + crops (tomato, chickpea, cowpea etc.); foliar spray, occasional pollen exposure	Weeks-long protection; specific target knock-down; compatible with standard spraying	GH → pre-field; highest TRL among nano-RNA platforms
Conceptual nano-precision breeding	Yield (*Gn1a*, *DEP1*), S-genes (*MLO*, *SWEET*), stress regulators	CNTs, MSNs, polymer NPs, LDH NPs, DNA nanostructures	CRISPR RNPs, base/prime editors, regulatory RNAs	Rice, wheat, maize, legumes (envisaged); meristems, floral tissues, seeds	No published heritable nano-mediated edits at major loci; concept is tissue-culture-free editing of elite lines	Concept only/early lab (TRL 1–2)

*Row kept to anchor SWEET/mlo genetics; explicitly marked as no nano yet to avoid confusion.

*TRL, technology readiness level; PoC, proof-of-concept; GH, greenhouse; BLB, bacterial leaf blight; NPs, nanoparticles; SWNTs, single-walled carbon nanotubes; MSNs, mesoporous silica nanoparticles; LDH, layered double hydroxide; CNTs, carbon nanotubes.

### 6.4 Interdisciplinary collaboration

Climate change, environmental degradation, and increasing population is adversely affecting agriculture due to rising temperatures, abrupt patterns of precipitation, and increasing levels of CO_2_. Mitigation of these multifaceted glitches requires collaborative roles of experts in the field of nanotechnology, plant breeding, plant physiology, and agronomy.

#### 6.4.1 Nanotechnologists

Recently, agricultural nanotechnology emerges as the field of research and innovation that integrates nanoscience and agricultural practices, with a great potential of promoting sustainable farming approaches (Balusamy et al., 2023; Abd-Elsalam, 2024). Nanotech engineers are specialized in synthesizing and applying nano materials and instruments, thus bringing innovations in modern agriculture. They can offer a collaborative role by developing agricultural inputs and tools such as nano fertilizers, nano pesticides, nano sensors, and targeted delivery systems. Farming practices, crop yield, and soi quality can be improved with the application of nanoproducts and nano technologies. Besides, this integrated approach paved a way for wide range of applications such as nanosensors and nano fertilizers to monitor plant health and ensuring efficient delivery of nutrients, respectively. Furthermore, nano-enabled agrochemicals are fruitful in targeted delivery and precise release that ensures efficient pest and disease management (Shraogi et al., 2025), thus mitigating the environmental risks associated with the use of conventional agrochemicals. On the other hand, nanotechnology is also crucial in precision agriculture by integrating nanoengineered sensors with Internet of Things (IoT) which aids in detecting early stress responses in plants (Wang, 2026; Gulia et al., 2025).

#### 6.4.2 Plant physiologists

Since nanotechnological applications are also associated with plant physiology and agronomy, plant responses to nanomaterials-based inputs and agronomic practices are well interpreted by physiologists who are capable of measuring photosynthetic changes, water-use efficiency, nutritional status, and stress-signalling pathways by applying nano products. For instance, the efficiency of chemicals use for priming (production of stress resistant plants) in plants can be enhanced by the application of nanotechnological tools in chemical priming, thus controlling chemical release into the environment (Gohari et al., 2024). In addition, combining plant physiology and nanotechnology is crucial in the production and response validation of biotic and abiotic stress tolerant crops.

#### 6.4.3 Breeders and agronomists

Similarly, recent advances in agricultural nanotechnology have also been expanded to plant breeding. Plant breeders use nano sensors for accurate measurement of physiological traits (photosynthetic rate, stomatal conductance etc.) and management of agronomic factors in plant progenies, such as planting density and irrigation practices. Moreover, plant breeders aim to convert genetically transformed plants into elite cultivars by ensuring trait stability across multiple generations and critically evaluating productivity, quality and adaptability of the subsequent generations (Swarup et al., 2021). From a translational perspective, scalability remains a critical bottleneck. Even where delivery and editing are demonstrated, extending these results to large populations of breeding lines under field relevant conditions remains unresolved.

## 7 Challenges, biosafety, and regulatory considerations

Regulatory treatment of genome-edited crops remains jurisdiction-specific. In the United States, FDA’s 2024 guidance frames foods from genome-edited plants within the existing risk-based new plant variety policy and voluntary premarket engagement framework, while USDA APHIS confirmed that the 2020 update to 7 CFR part 340 was prospectively vacated in December 2024, creating additional procedural uncertainty for plant movement and exemptions. In the European Union, the New Genomic Techniques proposal advanced to a provisional agreement in late 2025 and early 2026, indicating movement toward a separate framework for plants that do not contain foreign DNA. In China, the approval still depends on the biosafety evaluation and safety-certificate procedures under the agricultural GMO framework. For nanoparticle-enabled delivery, an extra level of uncertainty concerns the carrier material itself, its environmental persistence, and any residual material that may remain in the final product. Regulatory frameworks vary a lot from one region to another. The U.S. relies on a product-based method, whereas the EU is characterized by a more rigorous process-based regulation. In Asia, the regulatory systems differ with some countries like China having biosafety evaluation frameworks.

### 7.1 Nanotoxicity and environmental fate

Physicochemical interactions of engineered nanoparticles (ENPs) with soil, water, and biological matrices mainly determine the environmental fate of those ENPs which are used as carriers for genome-editing reagents. Over the last couple of years, research mainly shows that the toxicity and transport of these materials are not their inherent properties but are influenced by environmental aging and the formation of a biological identity.

Mechanistic categorization of nanoparticle-induced phytotoxicity can be put in two ways:

- Biochemical toxicity, largely linked to the generation of reactive oxygen species (ROS), ions release (e.g. metal ions from metallic nanoparticles), and oxidative stress signalling which leads to disruption of cellular homeostasis.
- Physical toxicity, which results from a situation where a nanoparticle physically blocks the plasmodesmata or stomatal pores, or the accumulation of nanoparticles in apoplastic spaces disrupts transport processes.

This distinction is especially important for directing “green synthesis” strategies because biochemical toxicity can be mitigated by modifying the surface and using inert materials, while physical toxicity depends on size, aggregation state, and tissue localization.

The moment a nanoparticle is introduced into an agricultural ecosystem, it will almost immediately experience matrix-dependent changes such as aggregation, dissolution, and sulfidation. Smaller nanoparticles, usually those in the sub-20 nm range that required to penetrate the cell wall, have a higher mobility and reactivity. As a result, not only can they be more easily translocated into the edible tissues of plants, but on the other hand, their risk of phytotoxicity potential is also increased. These types of phytotoxic impacts can be expressed as a reduced seed germination, a hampered root growth, and an oxidative stress resulting from the generation of reactive oxygen species (ROS) (Hatami and Ghorbanpour, 2024).

An important secondary finding from recent systems biology research is that of eco-corona formation. In natural waters or soil porewater, nanoparticles pick up surface coatings made of proteins, polysaccharides, and natural organic matter (NOM). This eco-corona essentially hides the synthetic identity and targeting ligands of the nanocarrier, altering the biological system’s recognition and processing of the particle. For example, acquisition of protein-rich corona can shift the dominant endocytosis pathway and this can change the intracellular trafficking and eventual degradation of the editing cargo Sooriyakumar et al. (2022). Eco-corona formation can drastically change nanoparticle behaviour. Significantly, this corona could help the co-transport of environmental contaminants into plant tissues e.g. heavy metals or organic pollutants. Although evidence in crop plants is limited, the issue of accidental uptake pathways and food safety is such an important concern that it definitely needs to be looked at further investigation.

Nanocarrier’s chemical makeup actually controls their environmental persistence. A number of noble metal platforms, among them gold nanoparticles (AuNPs), are chemically inert and hence environmentally persistent however they mostly cause low acute toxicity (Pourali et al., 2023). On the other hand, carbonic nanomaterials can be degraded by plant peroxidase, such as horse radish peroxidases (HRP is an example), which should actively shorten the tubes within a few weeks. Inorganic carriers such as mesoporous silica nanoparticles (MSNs) constitute a special case when they slowly hydrolyse convert to orthosilicic acid, which is a chemically definite dissolution path (Pourali et al., 2023). However, cationic surfactants, or dopants that are used to increase loading, may raise toxicity to non-target soil invertebrates and microbial communities, which is need for green synthesis and biodegradable scaffolds (Abd-Elsalam, 2024).

### 7.2 Regulatory landscape for nano-enabled genome editing

The regulatory status of genome-edited crops is currently changing, with major global agricultural producers becoming progressively more divergent. The main legal issue is whether nanoparticle-delivered genome editing should be regulated based on the presence of foreign DNA or the technology used to make the edit. Recently, the United States, China, and the European Union have made substantial modifications to their frameworks to handle “transgene-free” edited crops (Haider et al., 2026). As nanoparticle delivery opens up the possibility to introduce RNPs or transient mRNA without the integration of a stable transgene, theoretically many of these products would fall outside the traditional definitions of Genetically Modified Organism (GMO) (Tsanova et al., 2021). Genotype editing results are generally categorized as SDN-1, SDN-2, and SDN-3. SDN-1 means that there are small insertions or deletions without a repair template and it usually is considered transgene-free under various regulatory frameworks including the ones in the U.S. On the other hand, SDN-2 and SDN-3 are template-guided modification or insertion of larger sequences and they could be more heavily regulated especially in the European Union.

The USDA SECURE rule in the United States was suddenly put on hold when a federal court in late 2024 vacated it. The court decided that the rule did not fulfil certain specific procedural requirements, which led to the regulatory environment being restored to how it was prior to 2020. The Food and Drug Administration (FDA) at the same time issued updated guidelines in 2024, explaining that food made from genome-edited plants will be, in principle, regulated with the same safety requirements as those applied to traditionally bred crops while highlighting a risk-based, voluntary pre-market consultation process (FDA, U, 2024; Loschin et al., 2025).

China has taken an increasingly proactive stance, viewing biotechnology as a foundation for national food security. Recently, China has approved safety certificates for multiple gene-edited varieties of soybean, wheat, and corn. In contrast, the European Union has historically treated all genome-edited crops as GMOs. However, a major policy shift occurred recently with the proposal of a “New Genomic Techniques” (NGT) framework. In 2024, the European Parliament voted to ease requirements for certain edits, and in 2025, the European Council endorsed a negotiating mandate to formalize these regulations (United States District Court for the Northern District of California, 2024).

### 7.3 Scalability and field translation

Field translation remains constrained by manufacturing reproducibility, cargo stability, and delivery mode, so the most scalable near-term uses are foliar RNAi applications and transient target validation rather than broad acre genome editing. Despite the precision offered by nanoparticle-based delivery in laboratory settings, achieving scalability for field-scale application remains a primary engineering bottleneck. Most laboratory protocols rely on syringe infiltration or vacuum infiltration, which are impractical for broad-acre agricultureThe industrial production of nanomaterials is encountered by issues regarding reproducibility, safety, and cost-effectiveness of scaled-up processes (Seder et al., 2024). The latest innovations in microfluidic synthesis have given rise to a potential way out of such challenges. Recently released Universal Flow Rate platforms are capable to work at very broad flow rate ranges (from 0.1 to 0.7 mL/min) without compromising of mixing capabilities, thus allowing the large-scale production of lipid nanoparticles (LNPs) and polyplexes with uniform size (Islam Seder et al., 2024).

Implementation of field translation always necessitates the optimization of modes of application in order to ensure that the nanoparticle-cargo complex gets to the targeted tissue effectively. Foliar spraying is the most scalable method, but it has to overcome barriers such as the leaf cuticle and stomatal resistance (Schwartz et al., 2020). Studies have recently shown that Supercritical Fluid Technology (SFT) can be used to prepare crop protection products with nano reinforcements, which have a very high surface-area-to-volume ratio, improving the penetration through plant’s structural barriers (Faisal et al., 2025). Also, moving away from tissue culture towards tissue-culture-free approaches, such as pollen magnetofection or direct meristem transformation, is essential for elite germplasm editing (Quiroz et al., 2025; Hu and Liu, 2025).

The cost-benefit analysis of these systems reveals that although initial R&D spending might be higher due to the complexity of nanocarrier engineering, the significant advances in the field of crop resilience may eventually cover the extra expenditure. Several genome-edited crops have already entered the market or received regulatory approvals, particularly for traits such as improved shelf life, nutritional enhancement, and industrial applications (Polidoros et al., 2024). However, the economic impact of the introduction of tariffs in 2025 on specialized enzymes and nucleic acid synthesis platforms has increased R&D costs, prompting seed companies to adjust their sourcing strategies.

### 7.4 Public perception and acceptance

Effective adoption of nano-enabled genome editing technologically relies on public trust and safety assurance. As per national survey reports, especially a comprehensive study in South Korea (n = 1055) by authors Oh and Lee published in 2025, the public attitude towards the subject has been unravelled significantly. A most interesting aspect in the study is regarding the terminology gap, while people are generally unaware of technical terms like “CRISPR”; however, they are aware of easily understandable terms like “gene scissors.” Although 70% who are ready to buy gene-edited products this is considerably more than the previous acceptance levels for GMO. Still, this acceptance heavily depends on scientific transparency.

Generally, public skepticism is because of the worry about corporate monopoly and also about the possibility of unintended ecological disruption. Perception differes totally different way, depending on the application domain; for instance, people are usually more open towards medical gene therapy than to agricultural genome editing (Oh and Lee, 2025). They also pointed out a “credibility-accessibility gap, “ meaning that even though the public trusts expert organizations, the main source of their information is mass media. In order to close this gap, one really needs to communicate clearly the benefits of nano-enabled precision breeding as a way of gaining societal acceptance (Shaheen et al., 2023; Balusamy et al., 2023).

## 8 Future perspectives

Near-term progress in nanoparticle-enabled precision breeding is most likely to come from improved synthesis control, plant-specific targeting, better cargo stability, and reproducible RNAi formulations. Mid-term advances may include validated machine-learning models trained on plant-specific uptake and delivery datasets. Long-term possibilities such as autonomous AI-guided nanocarriers, DNA-origami logic systems, and organelle-specific genome editing should be presented as hypotheses rather than expected outcomes. Terms such as AGILE and NanoSafari should be defined in one sentence on first use and retained only if the underlying sources are primary and traceable. Future developments in nanocarrier enabled plant genome editing can be broadly divided into near term improvements and longer-term speculative directions. Near term progress is likely to focus on improving delivery efficiency, tissue specificity, and reproducibility. In contrast, concepts such as fully autonomous smart nanocarriers or integrated AI driven design platforms remain exploratory and will require substantial experimental validation.

### 8.1 Smart and AI-guided nanocarriers

A major significant change in nanoparticle design is the shift from conducting experimental work through trial and error to using intelligent, data-driven approaches. Recently, Artificial Intelligence (AI) and Machine Learning (ML) have become very effective tools to rationalize nanocarrier formulation, predict biodistribution, and optimize design parameters (Eskandani, 2025). AI-powered platforms are being harnessed to screen through extensive nanomaterial libraries to derive structure-function relationships, thus reducing dependency on resource-intensive trial-and-error experiments (Eskandani, 2025). Structural data is also being analyzed using deep learning models to screen for high-affinity sequences (Dimple, 2025).

An interesting development is the AI-Guided Ionizable Lipid Engineering (AGILE) platform which leverages Graph Neural Networks (GNNs) for the optimization of lipid nanoparticle formulations by varying features such as pKa and phospholipid chain length to enhance endosomal escape (Eskandani, 2025). Moreover, Large Language Model (LLM)-based platforms such as “NanoSafari” are leveraging the knowledge from more than 20,000 scientific publications to offer personalized suggestions for delivery systems (Eskandani, 2025). Besides, generative networks like Generative Adversarial Networks (GANs) are being considered for the creation of new nanocarrier designs with improved stability (Eskandani, 2025).

On top of all that, the idea of “Digital Twins” in nanomedicine is becoming more popular. By integrating multi-omics data with cultivar-specific biomarkers, AI systems can customize delivery systems for each person (Eskandani, 2025). This AI-aided design also helps in simulating the nano-bio interface, especially the contact of nanoparticles with the complex carbohydrate network of the plant cell wall (Dimple, 2025). Machine learning models based on the nanoparticle physicochemical properties and plant cellular uptake rates have started to forecast how small variations in zeta potential or surface amination affect wall traversal with sub-10 nm precision (Dimple, 2025).

### 8.2 Precision organelle editing

With genome editing expanding beyond the nucleus, organelle engineering, especially chloroplast and mitochondrial genome modification, has become a major avenue for crop enhancement. Additionally, organelle editing carries a several advantages such as absence of gene silencing and maternally inheritance (Tsanova et al., 2021). Recently, nanotechnology has begun unlocking this potential by introducing multi-stage navigation systems that can target organelle matrices (Law et al., 2023).

Recent advances have highlighted the use of “smart” nanocarriers pre-assembled with specific navigational layers. These systems often utilize Navigation Layers consisting of targeting ligands such as chloroplast transit peptides or mitochondria-targeting Cytox peptides (Das Karmakar et al., 2025). A second Transport Layer, often composed of high-aspect-ratio carbon nanotubes (CNTs) or sub-10 nm clusters, facilitates entry through the cell wall barrier (Santana et al., 2022). Finally, a Release Layer incorporating pH-responsive or reduction-responsive “gates” on mesoporous silica nanoparticles ensures the cargo is released only once it reaches the target organelle (Santana et al., 2022).

Mitochondrial engineering is particularly significant for enhancing agricultural productivity in energy crops (Das Karmakar et al., 2025). The integration of CRISPR-Cas systems with nanotechnology is also improving prime editing (PE) systems; for instance, advanced prime editing (ePPEplus) in hexaploid wheat has achieved the multi-locus and multigene editing required for fine-tuning complex agronomic traits.

### 8.3 Climate-resilient agriculture

The urgent requirement for sustainable agriculture in the face of climate change is driving the convergence of nanotechnology and crop physiology. Recently, nano-enabled “stress-smart” agriculture has offered innovative solutions to enhance nutrient use efficiency (NUE) and resilience to abiotic stresses (Khan et al., 2026; Raza et al., 2023). Nanocarriers improve the delivery and stability of natural hormones and biostimulants (Khan et al., 2026).

Evidence from field trials and meta-analyses published in the past couple of years indicates that nano-enabled fertilizers and biostimulants can achieve yield gains of 15% to 35% while reducing chemical usage by up to 40% (Khan et al., 2026). For instance, Layered Double Hydroxide (LDH) “BioClay” nanosheets have been shown to provide weeks-long virus protection via topically applied dsRNA, significantly outperforming naked dsRNA. In the context of abiotic stress, recently, silicon nanoparticles (SiO2 NPs) have been shown to be an effective means of reducing salt stress in rice plants by regulating ion homeostasis (Xiong et al., 2024). Additionally, selenium and silver nanoparticles were discovered to modify plant physiological responses under temperature stress by enhancing hydration.

The future of climate adaptation lies in integrating these nano-enabled inputs with advanced genome editing to create crops with “programmed” resilience. By using CRISPR to modify target genes such as *DREB* and *NAC* transcription factors and delivering these tools via nanocarriers, breeders can introduce favorable alleles into adapted local germplasm while maintaining essential genetic diversity (Awan et al., 2024).

### 8.4 Convergence with synthetic biology and systems biology

The ultimate frontier of plant engineering is the convergence of nanotechnology with synthetic biology and systems biology. Synthetic biology offers the rational design of biological systems with programmable functions, such as synthetic carbon fixation cycles or microbiome-engineered soil carbon sequestration. Nanotechnology serves as the critical delivery interface for these complex genetic constructs. The Design Build Test Learn (DBTL) cycle is being accelerated recently by the use of nanomaterials that allow for high-throughput, non-integrative trait testing in mature plants (Cai et al., 2025; Orsi et al., 2026).

Systems biology provides the necessary understanding of how nanomaterials interact with the plant’s molecular networks. Recent research has focused on the synergistic interactions between nanoparticles and beneficial microorganisms to optimize plant growth under stress conditions (Abd-Elsalam, 2024). For example, nanoparticles can be used as carriers for microbial inoculants, ensuring targeted delivery to plant roots (Abd-Elsalam, 2024). Advanced biosensors and microfluidic platforms are also being developed to monitor plant-microbe dynamics in real-time, offering a “smart farming” approach that integrates diagnostic feedback with therapeutic action (Abd-Elsalam, 2024).

In the coming years, the development of hybrid and multimodal systems—such as lipid-coated DNA origami or polymeric cores decorated with targeting aptamers—will likely herald a new era of precision nanomedicine in agriculture (Eskandani, 2025). Once these systems become responsive, they will have the ability to utilize real time biological data to guide genome editing or nutrient release precisely at the time and place where the plant needs it (Abd-Elsalam, 2024; Eskandani, 2025).

## 9 Conclusion

Nanoparticle design is moving towards more complex and sophisticated technologies convergence. Conventional material systems like LNPs and polymeric NPs are being regularly fine-tuned through advanced manufacturing technologies and computational modelling.

At the same time, new platforms such as DNA origami are providing unprecedented levels of programmability and functionality. The future belongs to hybrid and multimodal systems that integrate the strengths of different materials for instance, a lipid-coated DNA origami structure or a polymeric coreadorned with targeting aptamers and contrast agents.

Major issues remain like producing Good Manufacturing Practice (GMP) products at a truly large-scale and cost effective, knowing more about nano-bio interactions to predict their fate and toxicity in the long run, and enhancing targeting efficacy even beyond the limitations of the EPR effect. While solving these problems, the new generation of nanoparticles will probably be self-responsive, capable of incorporating diagnostic feedback to direct therapeutic action in real-time, thus announcing a new era of precision nanomedicine.

Nanoparticle-based precision breeding’s advanced delivery systems can greatly reduce dependence on chemical inputs in agriculture, as well as improve the plants’ immune ability to survive different environmental stresses. Efficient genetic cargo transport achieves the dual goals of optimizing resource, utilization and minimising the environmental footprint, along with accelerating the breeding of climate-resilient crops, thereby contributing to the sustainability of the agricultural domain. Collectively, current evidence positions nanocarrier enabled genome editing as a promising but still developing approach, with immediate applications in target validation and transient assays rather than direct field level deployment.
